# Targeting Cellular Metabolism in Acute Myeloid Leukemia and the Role of Patient Heterogeneity

**DOI:** 10.3390/cells9051155

**Published:** 2020-05-07

**Authors:** Ida Sofie Grønningsæter, Håkon Reikvam, Elise Aasebø, Sushma Bartaula-Brevik, Tor Henrik Tvedt, Øystein Bruserud, Kimberley Joanne Hatfield

**Affiliations:** 1 Department of Clinical Science, University of Bergen, 5021 Bergen, Norway; ida.gronningseter@uib.no (I.S.G.); Hakon.Reikvam@uib.no (H.R.); Elise.Aasebo@uib.no (E.A.); sushma.bartaula@uib.no (S.B.-B.); tor.henrik.anderson.tvedt@helse-bergen.no (T.H.T.); 2 Department of Medicine, Haukeland University Hospital, 5021 Bergen, Norway; 3 Department of Immunology and Transfusion Medicine, Haukeland University Hospital, 5021 Bergen, Norway

**Keywords:** leukemia, glycolysis, metabolism, oxidative phosphorylation

## Abstract

Acute myeloid leukemia (AML) is an aggressive blood cancer resulting in accumulation of immature, dysfunctional blood cells in the bone marrow. Changes in cell metabolism are features of many cancers, including AML and this may be exploited as a therapeutic target. In this study we investigated the in vitro antileukemic effects of seven metabolic inhibitors that target different metabolic pathways. The metabolic inhibitors were tested on AML cells derived from 81 patients using proliferation and viability assays; we also compared global gene expression and proteomic profiles for various patient subsets. Metformin, 2DG, 6AN, BPTES and ST1326 had strong antiproliferative and proapoptotic effects for most patients, whereas lonidamine and AZD3965 had an effect only for a minority. Antiproliferative effects on AML cells were additive when combined with the chemotherapeutic agent AraC. Using unsupervised hierarchical clustering, we identified a strong antiproliferative effect on AML cells after treatment with metabolic inhibitors for a subset of 29 patients. Gene expression and proteomic studies suggested that this subset was characterized by altered metabolic and transcriptional regulation. In addition, the Bcl-2 inhibitor venetoclax, in combination with 2DG or 6AN, increased the antiproliferative effects of these metabolic inhibitors on AML cells. Therapeutic targeting of cellular metabolism may have potential in AML, but the optimal strategy will likely differ between patients.

## 1. Introduction

Acute myeloid leukemia (AML) is the most common leukemia in adults. It is an aggressive hematologic malignancy characterized by accumulation and enhanced cell proliferation of immature myeloid precursors in the bone marrow, leading to marrow failure [1]. AML is a heterogeneous disease with regard to its phenotypic, molecular and clinical features, with variations in differentiation, karyotype, molecular genetics and epigenetics of the leukemic cells. There is also heterogeneity with respect to clinical chemosensitivity, that is AML-free survival after intensive induction and consolidation chemotherapy [2]. Current treatment is based on high-intensity induction chemotherapy; however, not all patients are eligible for this potentially curative treatment and a large amount of AML patients will relapse, which is associated with impaired survival. The 5-year overall survival rate after intensive therapy is below 50% and even lower for patients older than 70 years [3] and new treatment options for AML are therefore warranted.

Changes in cellular metabolism have been described as hallmarks of cancer and metabolic targeting is considered as a possible therapeutic strategy in various cancers, including AML [4]. Cancer cells are often more dependent on glycolysis and have increased energy requirements in comparison to normal cells that mainly employ oxidative phosphorylation (OXPHOS) to gain energy in the presence of oxygen [5,6]. The ability of malignant cells to alter their energy metabolism to meet their bioenergetic demands is crucial for cancer development [7]. Complex metabolic capacities may contribute to the aggressiveness of AML, allowing cells to survive under higher metabolic demands. However, AML patients are heterogeneous with regard to metabolic regulation [8,9,10,11,12] and in this study we attempt to further investigate if patients are heterogeneous also with regard to the antileukemic effects of metabolic targeting.

Several metabolic pathways can be utilized by cancer cells to gain energy and building blocks for growth and survival, including oxidative phosphorylation, glycolysis, glutaminolysis, fatty acid β-oxidation, the pentose phosphate pathway and lactate metabolism [13]. Hematological malignancies seem to rely on different metabolic routes depending partly on their genetic abnormalities [14,15,16,17,18,19], an observation suggesting that AML patients can be subclassified based on their metabolic phenotype and may vary in their susceptibility to various strategies for metabolic targeting. In the present study, we investigated the in vitro effects of seven metabolic inhibitors that affect various metabolic regulators/pathways in AML cells derived from a large group of consecutive patients, including metformin that activates the AMP-activated protein kinase (AMPK); 2-deoxy-d-Glucose (2DG), lonidamine and AZD3965 which can inhibit various steps in or influencing glycolysis; bis-2-(5-phenylacetamido-1,3,4-thiadiazol-2-yl) ethyl sulfide (BPTES) that inhibits glutaminolysis; 6-aminonicotinamide (6AN) that inhibits the pentose phosphate pathway and ST1326 that inhibits fatty acid metabolism (for detailed information see Table S1). Several of these agents have also been reported to inhibit oxidative phosphorylation (i.e., metformin and lonidamine). The aim of our study was to compare the effects of these seven metabolic inhibitors that target various metabolic pathways and thereby investigate whether leukemic cells from various patients differ in their susceptibility to the inhibitors.

## 2. Materials and Methods

### 2.1. Primary Human Cells

#### 2.1.1. Primary Human AML Cells

This study was approved by the Regional Ethics Committee (REK III 060.02, (100602), REK Vest 2013-634 (190313), REK Vest 2015/1410 (190615), 2017/305 (070417) and samples were collected after written informed consent. The main characteristics of the 81 consecutive patients are presented in Table 1; acute promyelocytic leukemia (APL) patients were excluded. Peripheral blood mononuclear cells were isolated from patients with at least 80% AML cells among blood leukocytes and generally a white blood cell (WBC) count >15 × 10^9^/L. Enriched AML cell populations could then be prepared by density gradient separation alone (density of 1.077, Ficoll-Hypaque; Nycomed, Oslo, Norway), and included >90% AML cells and contaminating cells were mainly small lymphocytes [20,21,22]. Primary AML cells were cryopreserved in RPMI 1640 medium (Sigma-Aldrich, St. Louis, MO, USA) with 10% dimethyl sulfoxide and 20% heat-inactivated fetal bovine serum (FBS) and stored in liquid nitrogen until thawed and used in experiments.

#### 2.1.2. Primary Umbilical Cord Blood (UCB) Cells

Cells were obtained from five donors after written informed consent (REK Vest 2015/1759 (051115), 2017/305 (070417)). Mononuclear cells were enriched in a similar manner to AML cell populations, by using density gradient separation and stored in liquid nitrogen until used in experiments.

### 2.2. Reagents

Culture medium used in all experiments, except the coculture studies, was serum-free Stem Span SFEM^TM^ medium (Stem Cell Technologies, Vancouver, BC, Canada) supplemented with exogenous granulocyte-colony stimulating factor (G-CSF), stem cell factor (SCF) and fms-related tyrosine kinase 3 ligand (FLT3L). All cytokines were purchased from Peprotech (Rocky Hill, NJ, USA) and used at a final concentration of 20 ng/mL. The seven agents tested on AML cells are considered inhibitors that target metabolism and are described in Table 2. Metformin hydrochloride (PHR1084), 2-Deoxy-d-Glucose (abbreviated 2DG; 154-17-6), lonidamine (L4900) and bis-2-(5-phenylacetamido-1,3,4-thiadiazol-2-yl) ethyl sulfide (abbreviated BPTES; SML0601) were all obtained from Sigma-Aldrich (St. Louis, MO, USA), while 6-aminonicotinamide (abbreviated 6AN; 100-093-15) and AZD3965 (199-12) were purchased from Cayman Chemical (Ann Arbor, MI, US), ST1326 (870853; also known as Teglicar) was purchased from Avanti Polar Lipids (Alabaster, AL, USA) and venetoclax (6960; also known as ABT-199) was obtained from Tocris Bioscience (Bio-Techne; Minneapolis, MN, USA). AraC (Cytosine Arabinoside) was provided by Pfizer (New York, NY, USA). The same lot numbers of inhibitors were used in all experiments. Stock solutions were prepared according to the distributors’ datasheets. Metformin and 2DG were passed through a 0.2 µm filter after being dissolved and all agents were aliquoted and stored at −20 °C. Aliquots were thawed only once and diluted with their respective solvents, either dimethyl sulfoxide (DMSO) or phosphate-buffered saline (PBS).

### 2.3. Analysis of Cell Proliferation, Viability and Constitutive Release of Mediators

#### 2.3.1. AML Cell Proliferation

Proliferation was analyzed using a [^3^H]-thymidine incorporation assay [23]. Cells were seeded (5 × 10^4^ cells/well, 200 μL medium/well) in flat-bottomed 96-well microtiter plates (Nuncleon^™^; Nunc, Roskilde, Denmark). Cell cultures were added metabolic inhibitors at various concentrations, medium controls and solvent/vehicle controls. After six days of incubation at 37 °C in a humidified atmosphere of 5% CO_2_, [^3^H]-thymidine (37 kBq in 20 μL saline per well; TRA 310; Amersham International, Amersham, UK) was added and cultures were incubated for an additional 22 hours before nuclear incorporation was determined by liquid scintillation counting. Proliferation (uptake of thymidine) was estimated by comparing values in treated cultures with untreated control cultures (cells seeded in medium alone) and detectable proliferation was defined as at least 1000 counts per minute (cpm). The median of triplicate cultures was used in all calculations.

#### 2.3.2. Cocultures of Human Mesenchymal Stem Cells (MSCs) and AML Cells

Human MSCs (Lonza, Cambrex BioScience, Walkersville, MD, USA) derived from a healthy donor (MSC24539) were expanded in complete MSC growth medium (MSCGM™; Lonza) with 10% heat-inactivated FBS and 4 mM L-glutamine. The MSCs were trypsinized and used in coculture experiments in passage four. AML-MSC cocultures were prepared in MSC medium and the cells were then seeded into transwell plates (Costar 3401 plates; Costar, Cambridge, MA, USA) separated by a semipermeable membrane (0.4 µm pore size), allowing only cytokine-mediated crosstalk between cells with no direct MSC-AML cell contact. The proliferation of both MSCs and AML cells were also analyzed using the [3H]-thymidine incorporation assay after three days of coculture, as described in detail previously [24].

#### 2.3.3. Viability

The percentages of viable, apoptotic and necrotic AML cells were determined by flow cytometry using the Apoptest™–FITC kit according to the manufacturer’s protocol (NeXins Research, Kattendijke, the Netherlands) [25]. Briefly, primary cells were cultured for 48 h in Stem Span SFEM™ medium supplemented with cytokines (1 × 10^6^ cells/mL in 1 mL/well) in 24-well culture plates with or without metabolic inhibitors. Cells were double stained with AnnexinV fluorescein isothiocyanate (FITC) and propidium iodide (PI) according to the distributor’s staining protocol. Acquisition of data was done using a FACSVerse™ flow cytometer (BD Biosciences, San Jose, CA, USA), with a minimum of 10,000 events collected for each sample. The flow cytometry data was analyzed using FlowJo v10.3 software (Tree Star, Inc., Ashland, OR, USA).

#### 2.3.4. Soluble Mediator Release

AML cells (1 × 10^6^/mL, 1 mL/well) were cultured for 48 h in Stem Span SFEM^™^ medium supplemented with G-CSF, SCF and FLT3L (1 × 10^6^ cells/mL and 1 mL per well) in 24-well culture plates (Nucleon^™^; Nunc, Roskilde, Denmark), before supernatants were harvested and stored at −80 °C until analyzed using human magnetic Luminex multiplex assays (LXSAHM-17, R&D Systems; Minnesota, MN, USA). Supernatants from cocultures of MSCs and AML cells were also harvested and analyzed with the Luminex multiplex assays. Nineteen analytes were measured—(i) the chemokines CXCL1, CXCL10, CXCL5, CXCL8/IL-8, CCL2, CCL3, CCL4, CCL5, (ii) the interleukins (ILs); IL-1β, IL-1RA, IL-6, (iii) the matrix metalloproteinases (MMPs); MMP-1, MMP-2, MMP-9, (iv) tumor necrosis factor-α (TNF-alpha), (v) the growth factors granulocyte-macrophage colony-stimulating factor (GM-CSF) and hepatocyte growth factor (HGF) and (vi) the proteinase inhibitors cystatin C and serpin E1/PAI-1.

### 2.4. Mutational Profiling, Global Gene Expression Profiling and Proteomic Analysis

#### 2.4.1. Mutational Profiling

The TruSight Myeloid Sequencing Panel (Illumina, San Diego, CA, USA) was used to assess 54 genes frequently mutated in myeloid leukemias as described previously [26] and data from this analysis was available for 35 out of 81 AML patients included in the study.

#### 2.4.2. Global Gene Expression Profiling

Analysis of global mRNA profiles was performed on samples derived from 21 patients using the Illumina iScan Reader based on fluorescence detection of biotin-labeled cRNA as described previously [26]. For each sample, 300 ng of total RNA was reversely transcribed, amplified and biotin-16-UTP-labeled (Illumina TotalPrep RNA Amplification Kit; Applied Biosystems/Ambion, USA). The quantity and quality of the biotin-labeled cRNA was assessed using the NanoDrop spectrophotometer and Agilent 2100 Bioanalyzer, respectively. Biotin-labeled cRNA (750 ng) was hybridized to the HumanHT-12 V4 Expression BeadChip that targets 47,231 probes mainly derived from genes in the NCBI RefSeq database (Release 38). Data from the array scanning were investigated in GenomeStudio and J-Express 2012 [27]. All arrays within each experiment were quantile normalized before being compiled into an expression profile data matrix. The PANTHER version 14.1 was used for functional classification of genes [28,29].

#### 2.4.3. Proteomic Analyses

Proteomics preparation and analysis was performed on samples from 14 patients, as described previously [30,31]. Briefly, filter-aided sample preparation (FASP) procedure [30] was used to prepare samples, which were further analyzed on the Q Exactive HF Orbitrap mass spectrometer (MS) (Thermo Fisher Scientific; Waltham, MA, USA) coupled to an Ultimate 3000 Rapid Separator liquid chromatography (LC) system (Thermo Fisher Scientific). The LC-MS raw files were searched against a Swiss-Prot Homo sapiens FASTA file (downloaded 28.08.18) and the label-free quantitation (LFQ) count was set to 1 in MaxQuant version 1.6.1.0 [32].

### 2.5. Statistical Analyses

Statistical Package for the Social Sciences v. 23.0 (IBM SPSS statistics Inc., Chicago, IL, USA) and GraphPad prism v. 5.02 (Graph Pad Software, San Diego, CA, USA) software were used for statistical analyses. Mann-Whitney *U*-test, Kruskal-Wallis *H*-test with Dunn´s post hoc test for multiple comparisons, Wilcoxon signed-rank test, Welch’s *t*-test and Kendall’s test were used for statistical analyses. Differences were regarded as statistically significant when *p* < 0.05. J-Express 2012 was used to perform all cluster-analysis; all cluster-analysis were made using complete linkage. The statistical analyses used for proteomics analysis have been described elsewhere [31]. Significantly different proteins were analyzed in the STRING database (version 11.0) [33] using experiments and databases as interaction sources at a confidence cut-off score of 0.7 and visualized in Cytoscape (version 3.3.0) [34]. Since data was collected over multiple days, a data normalization step was performed to correct inter-day and plate variation. Essentially, this was done by registering the medians to equal one (1.00) and normalizing each data proportionally, termed “block correction.”

## 3. Results

### 3.1. Dose-Response Screening Studies of AML Cell Proliferation after Exposure to Metabolic Inhibitors

The [^3^H]-thymidine incorporation assay was used for the initial dose-response studies, that is we evaluated the ability of AML cells to survive seven days and still be able to proliferate after inhibitor treatment. Six concentrations of each inhibitor were initially tested (Figure S1). Metformin (1.25–40 mM), 2-DG (0.625–20 mM) and 6AN (50–500 μM) showed strong antiproliferative effects for all 17 patients investigated. The other four inhibitors had divergent effects and especially lonidamine (25–300 μM) and AZD3965 (6.25–200 nM) showed no or minor antiproliferative effects. Based on these initial results, the following inhibitor concentrations were chosen for further experiments—metformin 1.25 and 2.5 mM, 2DG 0.3 and 0.6 mM, 6AN 50 and 100 µM and BPTES 10 and 20 µM. Lonidamine and AZD3965 were further tested at their highest screening concentration of 300 µM and 200 nM, respectively, which were the maximum achievable concentrations in DMSO without having a toxic effect on AML cells. ST1326 was also further tested at its highest screening concentration of 50 µM; higher concentrations were not tested due to issues with poor solubility. AraC (0.0125–0.3 µM) had overall significant antiproliferative effects at all concentrations tested, with responses differing among patients (data not shown). Both DMSO and PBS alone showed no effect on AML cell proliferation or viability at the concentrations used in this study (data not shown).

### 3.2. Several Metabolic Inhibitors Have Antiproliferative Effects on Primary AML Cells, with no Association with Secondary AML, AML cell Differentiation, Karyotype, FLT3 or NPM1 Mutations, or Patient Survival

**Figure 1:**
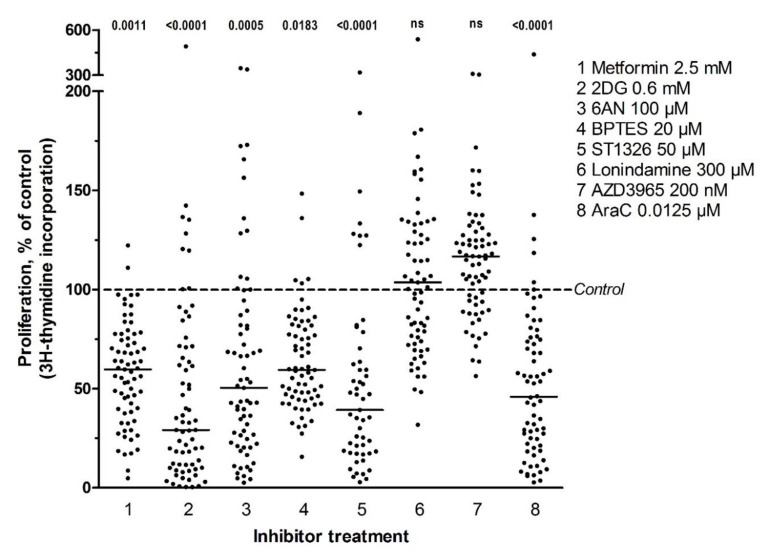
The effect of treatment with metabolic inhibitors or AraC on AML cell proliferation. AML cells were cultured alone in medium supplemented with cytokines or treated with either metabolic inhibitors or AraC for 7 days before proliferation was measured using the [^3^H]-thymidine incorporation assay. The inhibitor-free (untreated) controls were set to 100% (stippled line). Detectable proliferation was defined as >1000 cpm, which was found for 69 out of the 81 control cultures; while 62 cultures were treated with ST1326 whereof 53 control cultures had detectable proliferation. Each dot represents the percent proliferation compared to respective control cultures (with detectable proliferation) for one patient, with the median value for all patients indicated by the solid line. Statistically significant effects (*p*-values) are shown at the top of the figure (ns, not significant). The Kruskal Wallis with Dunn’s post-hoc test was used for statistical analyses.

We investigated the effects of metabolic inhibitors on cytokine-dependent AML cell proliferation for all 81 patients. Detectable proliferation, defined as [^3^H]-thymidine incorporation >1000 cpm, was observed in the control cultures for 69 of the 81 patients (85% of patients). For these patients with detectable cell proliferation, inhibitor treatment resulted in a highly significant antiproliferative effect compared to untreated controls (*p* ≤ 0.0001, Kruskal Wallis test, Dunn´s post-hoc test), for all inhibitors except lonidamine and AZD3965. The latter two inhibitors did not show any significant inhibitory effects on proliferation, though proliferation was decreased after inhibitor treatment for certain patients (Figure 1, Figures S2 and S3). When comparing the effect of inhibitors on proliferation for individual patients, we observed that the antiproliferative effect varied considerably between patients. For certain patients, cell proliferation was inhibited completely by inhibitor treatment while other patients had no effect with the same inhibitor concentration. The strongest overall antiproliferative effect was seen after treatment with 2DG (median inhibitory effect 76%, Figure 1). Furthermore, when we defined the antiproliferative effect as > 20% inhibition compared to untreated controls, we could not find any associations between those samples showing antiproliferative effects (higher versus lower than 20% inhibition by metabolic inhibitors) and AML cell differentiation (FAB classification, CD34 expression), cytogenetics, *FLT3* or *NPM1* mutations or occurrence of previous disease (versus de novo AML). A few associations of borderline significance were detected (*p* < 0.05, Table S2). Due to the large number of comparisons and small sample numbers, we conclude that the antiproliferative effects show no significant associations in these analyses. The antiproliferative effects of all seven metabolic inhibitors were reproduced in an independent experiment for 11 of the 81 patients (data not shown).

Our patient cohort included 40 younger patients who completed intensive induction and consolidation therapy; 15 of these 40 patients remained in remission for a median of 105 months (range 39–164 months) whereas the others died from resistant/relapsed AML. The antiproliferative effects observed with the metabolic inhibitors did not differ between patients when comparing long-term survivors and patients dying from resistant leukemia (Table S2).

### 3.3. Metabolic Inhibitors Have Additive Antiproliferative Effects when Combined with AraC

**Figure 2:**
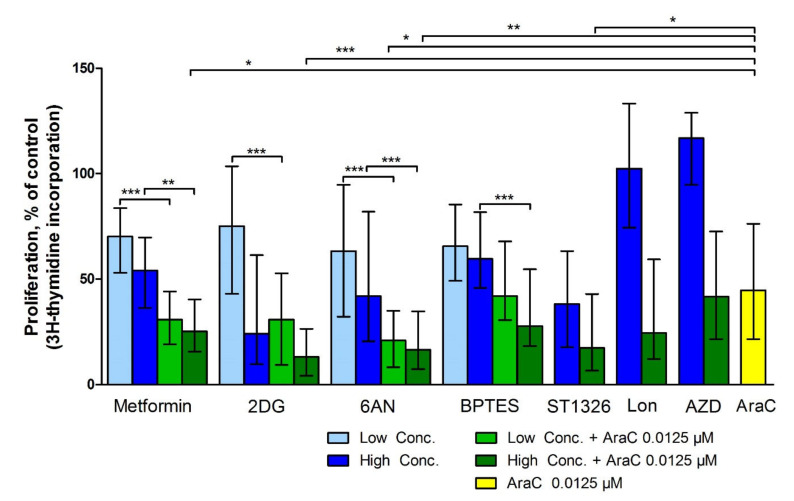
The effect of the combined treatment of metabolic inhibitors with AraC on AML cell proliferation, using the [^3^H]-thymidine incorporation assay. The metabolic inhibitors were tested at the following concentrations—metformin 1.25 and 2.5 mM, 2-DG 0.3 and 0.6 mM, 6AN 50 and 100 µM, BPTES 10 and 20 µM, ST1326 50 µM, lonidamine 300 µM and AZD3965 200 nM alone and in combination with AraC 0.0125 µM. All 69 patients with detectable proliferation in untreated control cultures were included in this analysis, except ST1326 which included 53 patients with detectable proliferation among 62 patients tested. The results are presented as the median percentage of proliferation in cell cultures treated with metabolic inhibitors alone or in combination with AraC compared to their corresponding control cultures, with interquartile range (whiskers). The Kruskal-Wallis test with Dunn’s post-hoc test was used for statistical comparisons between effects of metabolic inhibitors alone and metabolic inhibitors combined with AraC (significant *p*-values are shown right above each column) and between AraC alone versus combination treatment of AraC with metabolic inhibitors (illustrated at the top of the figure). * *p*-value < 0.05 and, ** *p*-value 0.001 and ***, *p*-value < 0.0001; Lon, Lonidamine; AZD, AZD3965.

The antiproliferative effects of metabolic inhibitors were also tested alone or in combination with AraC 0.0125 µM (81 patients examined, 69 showing detectable proliferation). The following concentrations of metabolic inhibitors were then used, metformin 1.25 mM and 2.5 mM, 2DG 0.3 and 0.6 mM, 6AN 50 and 100 µM, BPTES 10 and 20 µM, ST1326 50µM, lonidamine 300 µM and AZD3965 200 nM. Metabolic inhibitors combined with AraC had significantly greater antiproliferative effects compared to AraC alone, when testing metformin 2.5 mM, 2DG 0.6 mM, 6AN 50 and 100 µM and ST1326 50 µM (Figure 2 and Figure S4; *p*-value < 0.05, Kruskal Wallis test, Dunn’s post-hoc test).

### 3.4. The Antiproliferative Effect of Metabolic Inhibitors Differs between Patients and a Subset of Patients Show Increased Susceptibility to Several Inhibitors

**Figure 3:**
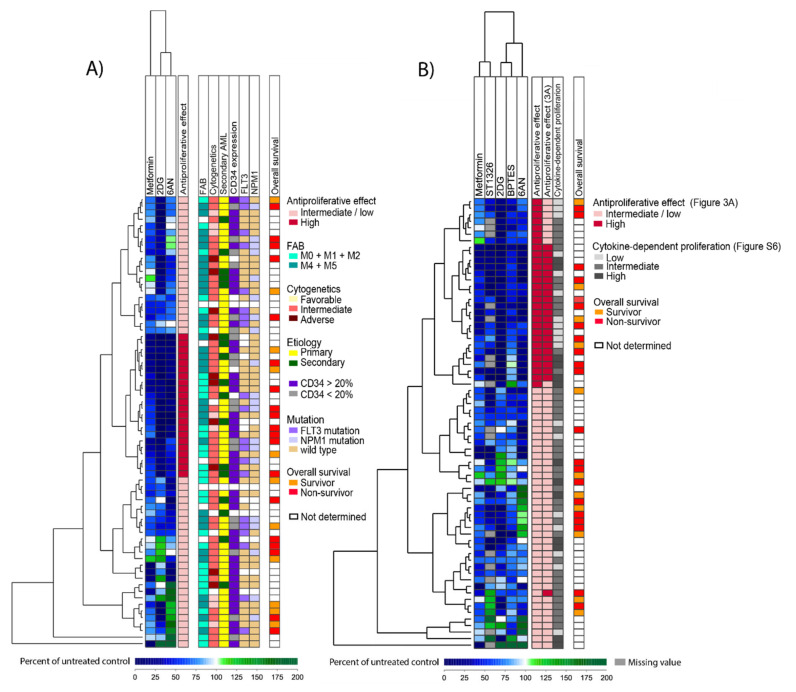
Unsupervised hierarchical cluster analysis based on the effects of metabolic inhibitors on proliferation of primary human AML cells. Primary AML cells were grown in medium supplemented with cytokines and treated with metformin 2.5 mM, 2DG 0.6 mM, 6AN 100 µM, BPTES 20 µM, ST1326 50 µM, lonidamine 300 µM and AZD3965 200 nM or medium alone (untreated controls) for 7 days before cell proliferation was analyzed using the [^3^H]-thymidine incorporation assay. The percent proliferation (i.e., proliferation in treated cultures relative to untreated controls) was estimated for each patient/inhibitor combination and patient subclusters are shown on the left side of each analysis. (**A**) Clustering based on effects of metabolic inhibitors that can influence glucose metabolism. The cluster analysis was based on the effects of metformin, 2DG and 6AN on AML cell proliferation and identified a subset of 22 patients with overall higher sensitivity towards these metabolic inhibitors (illustrated as dark red in the first column to the right). The next columns to the right indicate biological and clinical characteristics for each individual patient (FAB, cytogenetics, etiology, CD34 expression, *FLT3*-ITD and *NPM1* mutations, overall survival). (**B**) Clustering based on significant antiproliferative effects of metabolic inhibitors on AML cells. The cluster is based on the results for the five inhibitors that showed significant antiproliferative effects on AML cells when analyzing the overall results (see Figure 1). Two main patient subsets were then identified, the upper cluster included 29 patients (shown as dark red in the first column to the right) and showed stronger antiproliferative effects after inhibitor treatment, while the lower cluster included 40 patients (light red). Columns also visualize patient subsets identified in other cluster analyses, that is effects on proliferation after treatment with inhibitors influencing glucose metabolism (cluster data from Figure 3A) and effects on proliferation after treatment with all seven metabolic inhibitors (data from Figure S6) and overall survival.

One of the most prominent alterations in cancer cell metabolism is the increased consumption of glucose. We therefore did an unsupervised hierarchical cluster analysis based on the anti-proliferative effects of the metabolic inhibitors that can affect glucose metabolism, that is metformin, 2DG and 6AN (lonidamine was not included due to no significant effects on cell proliferation, Figure 1). This analysis was performed based on the percent inhibition of AML cell proliferation after treatment with inhibitors, that is the proliferation in treated cultures relative to the corresponding untreated control cultures. This clustering identified a subset of 22 patients with overall high sensitivity to these metabolic inhibitors. These 22 patients did not differ significantly from the other patients with regard to cause of AML (secondary versus *de novo*), AML cell differentiation (FAB classification, CD34 expression), karyotype, *FLT3*-ITD nor *NPM1* mutations, age, gender or survival of the younger patients receiving intensive therapy (Figure 3A).

We thereafter did an unsupervised hierarchical cluster analysis based on the five inhibitors with significant antiproliferative effects when analyzing the overall results (shown in Figure 1), which included metformin, 2DG, 6AN, BPTES and ST1326. We used only one concentration of each inhibitor that showed the widest range in antiproliferative effects between patients and we then identified two main clusters (Figure 3B). A subset of 29 patients (the upper cluster) was characterized by a strong antiproliferative effect by all or most of the five inhibitors (Table S3). These 29 patients did not differ significantly from the other patients with regard to cause of AML (secondary versus *de novo*), AML cell differentiation (FAB classification, CD34 expression), karyotype, *FLT3*-ITD, *NPM1* mutations or survival of the younger patients receiving intensive therapy (Figure 3B). Furthermore, the subset of 29 patients did not differ from the other patients with regard to degree of cytokine-dependent proliferation in control cultures.

The two hierarchical cluster analyses that were based on the antiproliferative effects of (i) inhibitors that can affect glucose metabolism (metformin, 2DG and 6AN) (Figure 3A) and (ii) the five metabolic inhibitors that had overall significant effects on cell proliferation (metformin, 2DG, 6AN, BPTES and ST1326) (Figure 3B), did not differ much with regard to patient distribution in the different subclusters. This shows that the results obtained with the three inhibitors metformin, 2DG and 6AN, in particular 2DG, weighed heavily in the clustering analyses.

Additional molecular genetic analyses were available for 35 patients (i.e., a subset of 35 consecutive patients from our cohort) and are summarized in Figure S5. Thirteen of these patients showed generally strong antiproliferative effects in response to metabolic inhibitors (Figure 3) whereas 17 patients showed weaker responses; both these groups showed a similar heterogeneity with regard to cytogenetic abnormalities and the frequency of patients with several molecular abnormalities did not differ between them. Twenty-eight genes of the 54 genes analyzed were mutated in at least one of the patients and most mutations had expected low frequencies [35]. The five metabolic inhibitors demonstrating significant overall effects on AML cell proliferation (i.e., percent inhibition after treatment compared to controls) showed no significant associations with any single mutation, a mutational class or the number of mutations per patient. Furthermore, when the gene mutations were classified based on the function of the encoded proteins (Figure S5), the frequency of mutations did not differ between the two patient subsets (identified in Figure 3B) for any of the mutational classes.

Finally, we performed an unsupervised hierarchical cluster analysis based on the effect of inhibitors on cell proliferation after values were normalized to their median values, when all metabolic inhibitors and all concentrations were included. Based on this analysis, the patients could be divided into three patient clusters (Figure S6), showing a generally high proliferation after treatment with inhibitors, low proliferation and divergent effects of inhibitors on proliferation. Patient samples with a relatively high cellular proliferation persisted to have high proliferation even after treatment with metabolic inhibitors, thus the observed variation of cellular proliferation between individual patients generally was maintained in the presence of inhibitors.

### 3.5. A Proteomic Comparison of Patient Samples Showing Either Generally Strong or Weak Antiproliferative Effects after Treatment with Metabolic Inhibitors

We did a proteomic analysis of AML cells derived from six patients where a generally strong antiproliferative effect was found after treatment with five metabolic inhibitors (Figure 3B, six patients among upper cluster of 29 patients), versus eight patients that showed either no or little effect of treatment (Figure 3B, lower cluster). The patient samples that were available for proteomic analysis represent a consecutive group of young and fit patients in our cohort receiving intensive antileukemic therapy. Forty-nine proteins showed a significant difference between the two groups (Welch’s t-test, *p* < 0.05) and thirty-one of these also had a significant fold change (*p* < 0.05) (Tables S4 and S5). Among these, the largest group of proteins belonged to the classification Metabolism (19 proteins, including 4 mitochondrial proteins) and/or were involved in transcriptional regulation (9 proteins). We also did a functional network analysis (STRING database v. 11.0) based on the 49 proteins that showed a significant difference only based on the Welch’s *t*-test (Table S6). We then identified 17 proteins that interacted with other significant proteins, where the majority of proteins were involved in the formation of a network of 12 proteins that are important for regulation of autophagy/metabolism and/or transcription (Table S6). Although this proteomic data and analyses should be interpreted with great care because few patients were included, they support that antiproliferative effects of metabolic inhibitors is influenced especially by the metabolic context (i.e., different expression of proteins involved in cellular metabolism) of the AML cells and in addition also by their transcriptional regulation.

### 3.6. A Comparison of Global Gene Expression Profiles for AML Samples that Differ in Their General Susceptibility toward Metabolic Inhibitors

Our proteomic analysis suggested that differences in the susceptibility to the metabolic inhibitors are associated with differences in metabolism and transcriptional regulation. We therefore compared the global gene expression profiles for 21 consecutive patients from our cohort, including eight patients with strong antiproliferative effects after inhibitor treatment (Figure 3B; among upper cluster of 29 patients) and 13 patients with intermediate/low antiproliferative effects (Figure 3B; lower cluster of 40 patients). We performed a feature subset selection (FSS) analysis and by selecting genes with an R-score > ±3.5, we identified 265 differentially expressed genes, 133 upregulated in the intermediate/low antiproliferative subset and 132 upregulated in the high antiproliferative subset. We then performed a hierarchical cluster analysis that clearly separated the two patient subsets (Figure S7). We thereafter performed gene ontology (GO) mapping using the PANTHER analytic tool based on the 265 differentially expressed genes. Among the categories Protein class and Biological process, we found several ontologies to be overrepresented in the two patient subsets. Genes belonging to transcription factors and involved in metabolic processes seem to be upregulated in the intermediate/low antiproliferative subset (cluster BI, Figure S7), while genes belonging to different enzyme systems (hydrolase, oxido reductase and transferase) and nucleic acid binding as well as cellular processes were upregulated in the subset with strong antiproliferative effects towards inhibitor treatment (cluster AII, Figure S7).

### 3.7. Metabolic Inhibitors Decrease AML Cell Viability through Proapoptotic Effects

**Figure 4:**
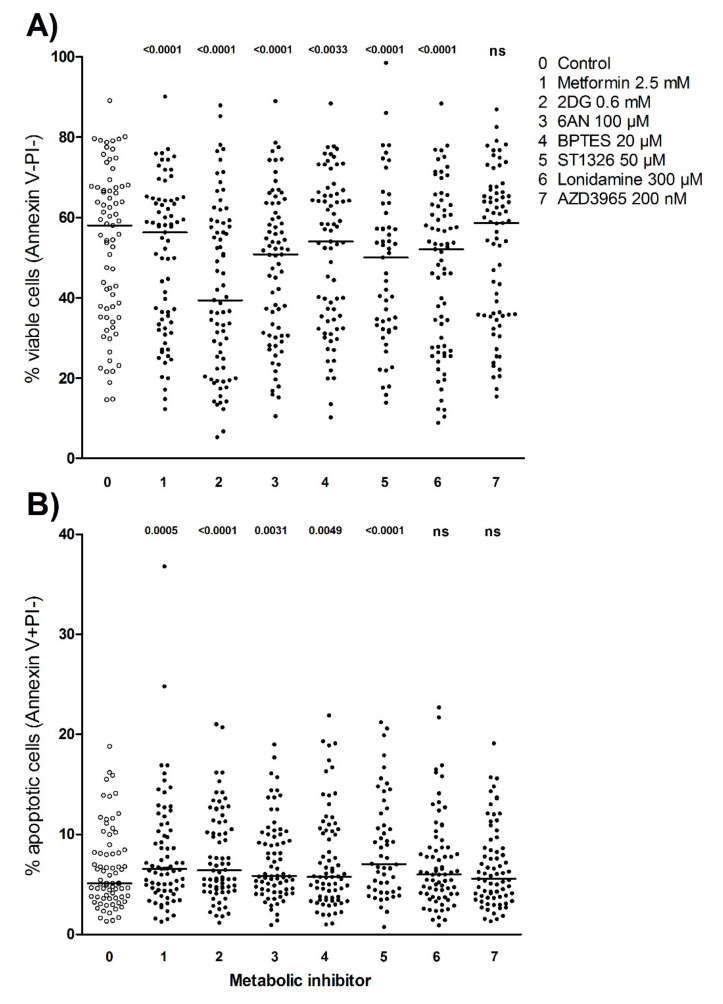
Effects of metabolic inhibitors on AML cell viability and apoptosis. AML cells derived from 78 consecutive patients were cultured for 2 days in suspension cultures in medium supplemented with growth factors with or without metabolic inhibitors, before the percentage of viable and apoptotic cells were determined by flow cytometry using the AnnexinV/PI apoptosis assay. Seventy-two patients had more than 5% viable cells in untreated control cultures and were used in the statistical analyses. The percentage of (**A**) viable and (**B**) early apoptotic cells for all 72 patients is presented. Each dot represents results for one patient sample, solid lines indicate median values. Significant effects (*p*-values) of inhibitor treatment compared to untreated controls are shown (Wilcoxon signed rank test; ns, not significant).

For 78 of the 81 patients, AML cell viability was analyzed after 48 hours of culture with or without treatment with metabolic inhibitors (three patient samples were excluded due to technical issues during the flow cytometric analysis). The highest concentrations of inhibitors that were tested in the proliferation assay, were also tested in the apoptosis flow cytometric assay, that is metformin 2.5 mM, 2DG 0.6 mM, 6AN 100 µM, BPTES 20 µM, ST1326 50 µM, lonidamine 300 µM and AZD3965 200 nM (Figure 4, Figure S8). The gating strategy to define viable, early apoptotic and late apoptotic/necrotic cells is illustrated for a representative sample in Figure S9; this gating strategy is the same as described in detail in a previous methodological study [25]. Of the 78 patients analyzed, six of the patients showed less than 5% viable cells in medium (untreated) controls and were excluded from the statistical analyses. The leukemic cell viability after 48 hours of culture showed a wide variation between patients, the median viability for untreated controls was 58% (range 14.6%-89.1%) and the median viability ranged from 39.4% after treatment with 2DG to 58.6% after treatment with AZD3965. All inhibitors except AZD3965 decreased AML cell viability compared to untreated controls for a significant majority of the patients (Wilcoxon signed rank test, *p*-value < 0.05) (Figure 4A). The percentage of apoptotic cells was generally relatively low after treatment with metabolic inhibitors for 48 hours; still, overall apoptosis was significantly increased by all inhibitors compared to untreated controls except for lonidamine and AZD3965 (Wilcoxon signed rank test, *p*-value < 0.05) (Figure 4B).

### 3.8. Identification of Patient Subsets Based on Effects of Metabolic Inhibitors on AML Cell Viability

**Figure 5:**
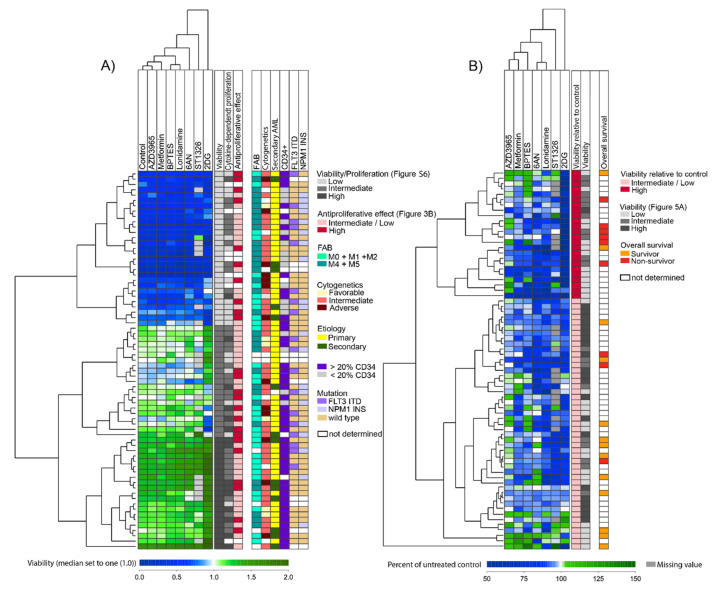
Unsupervised hierarchical cluster analysis based on the effects of metabolic inhibitors on AML cell viability. Primary AML cells were seeded into suspension cultures in medium supplemented with cytokines. The cells were treated with metformin 2.5 mM, 2DG 0.6 mM, 6AN 100 µM, BPTES 20 µM, ST1326 50 µM, lonidamine 300 µM and AZD3965 200 nM or untreated controls (medium alone) for 48 h before effects on cell viability were determined using the AnnexinV/PI flow cytometric assay. (**A**) Cluster analysis based on the viability of primary AML cells after treatment with metabolic inhibitors. The analysis is based on the percentage of viable cells in cultures after 48 hours with or without inhibitor treatment. The data obtained from the 72 patient samples were normalized to the corresponding median level (median set as 1.0). Patients could then be divided into three main subsets; one upper subset showing low viability (shown as white), one with intermediate viability (light grey) and one with generally high viability (dark grey) after inhibitor treatment. These subsets are indicated in the column to the right of the cluster, together with a column showing the degree of AML cytokine-dependent cell proliferation (based on Figure S6) and the effect of metabolic inhibitors on cell proliferation (based on Figure 3B), as well as biological and clinical characteristics for the individual patients (i.e., FAB classification, karyotype, etiology, CD34 expression, *FLT3*-ITD and *NPM1* mutations). (**B**) Cluster analysis based on the relative viability of AML cells after treatment with metabolic inhibitors for 48 h. The analysis is based on the relative effect of metabolic inhibitors on AML cell viability after 48 hours treatment, that is the viability in cell cultures treated with inhibitors relative to the viability in control cultures (untreated cultures set to 100%). Cluster analysis separated the 72 AML patients into two main subsets, one upper patient subset showing reduced viability after treatment with metabolic inhibitors (shown in dark red, 24 patients) and one lower subset with intermediate/low effects on viability (light red). The subsets are indicated in the column to the right of the figure, along with a column showing inhibitor effects on AML cell viability normalized to median levels (Figure 5A) and the classification of younger patients receiving intensive chemotherapy as survivors or non-survivors (i.e., dying from resistant/relapsed AML).

To further study the patient heterogeneity, we did a hierarchical cluster analysis based on AML cell viability after treatment with or without metabolic inhibitors (Figure 5A), where viability was normalized to corresponding median levels. We could then identify three patient subsets; an upper subset with a low cell viability after treatment with metabolic inhibitors (i.e., generally less viability than the median levels); while patients in the lower subset showed higher viability (i.e., generally higher than the median levels) after treatment with inhibitors, while the middle cluster showed divergent effects. Thus, the variation between patients with regard to spontaneous in vitro apoptosis is largely maintained in the presence of inhibitors targeting metabolic pathways.

We next did an unsupervised hierarchical cluster analysis based on the relative effect of the metabolic inhibitors on cell viability, that is percent viability of cell cultures treated with metabolic inhibitors compared to control cultures (cells in medium alone) and the clustering analysis then revealed two clusters (Figure 5B). An upper cluster including 24 patients was characterized by generally decreased viability (more apoptotic cells) after treatment with metabolic inhibitors and showed a relatively strong spontaneous in vitro apoptosis in control cultures. This patient cluster of 24 patients showed significantly decreased viability after treatment with 2DG (*p* < 0.0005) and 6AN (*p* = 0.013) compared to the other patient cluster (Table S7). Furthermore, the cluster with the strongest proapoptotic effect (decreased viability) after inhibitor treatment did not differ with regard to cause of AML (secondary versus *de novo*), cytogenetics, AML cell differentiation (FAB classification, CD34 expression), karyotype, *FLT3*-ITD or *NPM1* mutations. Finally, we could not detect any correlations between the antiproliferative and proapoptotic effects of individual inhibitors on patient samples except for 2DG, which showed a highly significant though weak correlation (Figure S10, *p* < 0.0005, Kendall’s τ = 0.321).

### 3.9. Effects of Metabolic Inhibitors on the Constitutive Release of Soluble Mediators By Primary AML Cells

**Figure 6:**
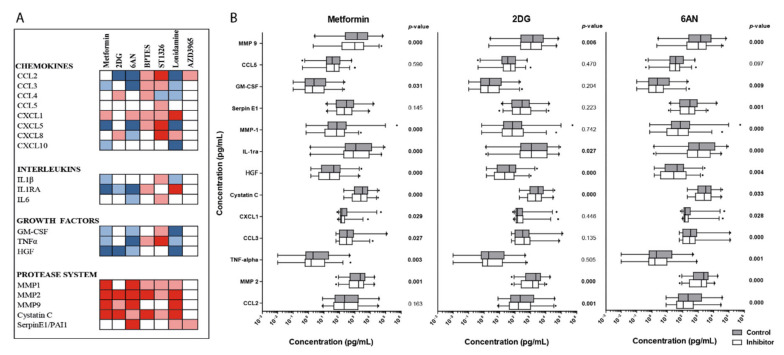
The effects of metabolic inhibitors on the constitutive release of soluble mediators by primary human AML cells. Primary AML cells from 72 patients were cultured for 48 hours with and without metabolic inhibitors before the concentrations of 19 soluble mediators were determined in supernatants using multiplex analysis. (**A**) Overall effects of metabolic inhibitors on the release of soluble mediators by AML cells. Concentrations were—metformin 2.5 mM, 2DG 0.6 mM, 6AN 100 μM, BPTES 20 μM, ST1326 50 μM, lonidamine 300 μM and AZD3965 200 nM. Mediator levels found in treated cultures were compared to untreated control cultures and increased levels after inhibitor treatment are shown in red, while decreased levels are shown in blue. Significant changes are indicated by the color grading (*p* < 0.05 light color, *p* < 0.001 dark color, Wilcoxon signed rank test). (**B**) Changes in the constitutive release of soluble mediators by metformin 2.5mM, 2DG 0.6 mM and 6AN 100 µM. Mediator concentrations measured in cultures after 48-hour treatment with metabolic inhibitors (white boxes) and untreated control cultures (grey boxes) are presented, with the median levels, 25/75 percentiles (boxes), 5/95 percentiles (whiskers) and outliers. The mediator concentrations are given on the X-axis as log-values. *P*-values are shown for significantly altered mediator concentrations when comparing treated cultures with untreated controls (Mann-Whitney *U*-test).

We analyzed the concentration of 19 soluble mediators in culture supernatants collected after 48 hours in vitro culture of AML cells derived from 72 patients. All patient samples analyzed had more than 5% viable cells in the control cultures after 48 hours of incubation (see above). The release profiles varied considerably between patients, however based on these profiles, patients could be clustered into three main subsets using hierarchical cluster analysis (Figure S11). Still, no significant associations were found between the release/levels of mediators and the effect of inhibitors on cell proliferation (Figure 3B) or cell viability (Figure 5B). Furthermore, the metabolic inhibitors had divergent effects on the release of soluble mediators (Figure 6A) that were relatively small compared with the large variations of mediator levels found between individual patients that were maintained in the presence of inhibitors (results shown for metformin, 2DG and 6AN in Figure 6B and Figure S12).

### 3.10. 2DG Has an Antiproliferative Effect on AML Cells Even in the Presence of AML-Supporting MSCs

**Figure 7:**
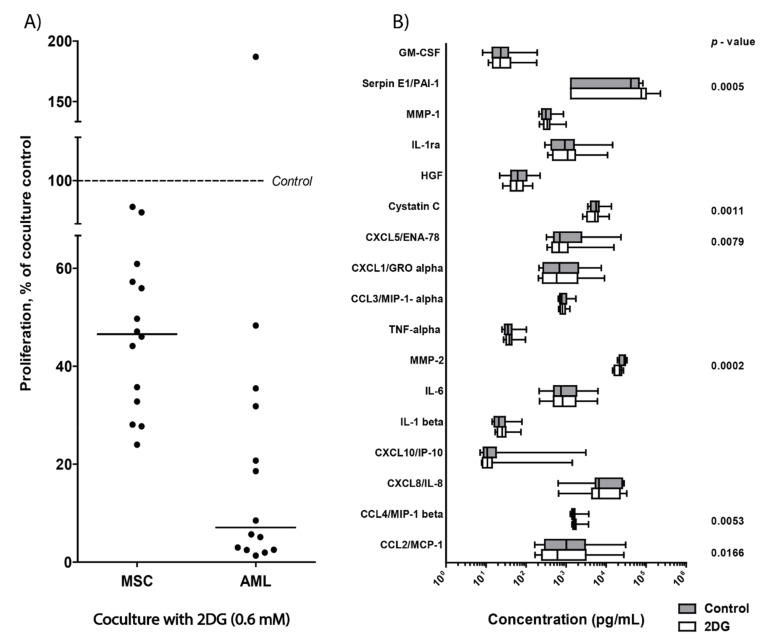
Cell proliferation and mediator release after in vitro coculture of primary human AML cells and normal mesenchymal stem cells (MSCs), after treatment with the glycolytic inhibitor 2DG. Primary AML cells derived from 14 patients were cultured together with normal MSCs in transwell cocultures for 3 days in the presence of 2DG, before proliferation was assayed using the [^3^H]-thymidine incorporation assay. Mediator levels were also determined in coculture supernatants with and without 48-hour treatment with 2DG, using multiplex analysis. (**A**) Effect of 2DG on cell proliferation in MSC-AML cocultures. Proliferation of MSCs or AML cells in 2DG-treated cocultures relative to the inhibitor-free control cocultures (shown as stippled line at 100%) was calculated and an antiproliferative effect by 2DG was then observed both for the MSCs (derived from one donor) and the AML cells (derived from 14 patients) during coculture. The median proliferation of the MSC cells and the AML primary cells after treatment in cocultures is indicated in the figure ( ‒ ). (**B**) The constitutive release of soluble mediators in cocultures after 48-hour treatment with 2DG. The results are presented as the median levels with 25/75 percentiles (boxes), 5/95 percentiles (whiskers). The mediator concentrations (pg/mL) are given on the X-axis as log-values. Significant *p*-values are shown to the right (Wilcoxon signed rank test; 2DG-treated cocultures compared to untreated cocultures). Grey boxes illustrate mediator levels in untreated cocultures and white boxes indicate levels after treatment with 0.6 mM 2DG.

2DG was the metabolic inhibitor with the strongest antiproliferative and anti-viability effects when analyzing the overall results (Figure 1 and Figure 4). Using the [3H]-thymidine incorporation assay, we therefore investigated whether 2DG (0.6 mM) also inhibited AML cell proliferation in the presence of normal MSCs when the different cell types were separated by a semipermeable membrane with no direct contact. Primary AML cells derived from 18 unselected patients (i.e., randomly selected from our patient cohort) were tested, while only 14 patients showed detectable AML cell proliferation in the medium controls. AML cell proliferation decreased after treatment with 2DG for most patients even in the presence of MSCs (Figure 7A). Furthermore, MSC proliferation was also decreased after 2DG treatment of cocultures (Figure 7A), though 2DG had a stronger antiproliferative effect on AML cells than on MSCs (Mann-Whitney *U*-test, *p* = 0.0016). The constitutive release of soluble mediators was also measured in cocultures with and without 2DG treatment. Only minor changes in mediator concentrations were found in 2DG-treated cocultures compared to control cocultures (cells cocultured in medium alone) and overall the mediator release profile seemed to remain similar in the absence and presence of the glycolytic inhibitor 2DG (Figure 7B, Figure S13).

### 3.11. Dose-Response Studies of Umbilical Cord Blood Cells Treated with Metabolic Inhibitors, Effects on Proliferation and Cell Viability

The effects of metabolic inhibitors on the proliferation of human UCB-derived mononuclear cells were evaluated using the [^3^H]-thymidine incorporation assay. Five samples where tested while four UCB samples showed detectable proliferation (>1000 cpm) in untreated control cultures. Treatment with metabolic inhibitors had a similar antiproliferative effect on UCB cells as seen for primary AML cells (Figure S1), however, metformin 5 mM inhibited proliferation of AML cells more than UCB cells (*p* = 0.030, Mann-Whitney *U*-test).

The effect of metabolic inhibitors on the viability of UCB-derived mononuclear cells was also analyzed by flow cytometry after 48 h of treatment with metformin 2.5 mM, 2DG 0.6 mM, ST1326 50 µM, BPTES 20 µM, AZD3965 200 nM and lonidamine 300 µM. The UCB cells from five donors were generally less sensitive towards all the metabolic inhibitors, with at least 90% viability after treatment compared to inhibitor-free controls (Figure S14).

### 3.12. Combination Treatment of Venetoclax and a Metabolic Inhibitor (2DG or 6AN) has a Stronger Antiproliferative Effect on AML Cells than Venetoclax or the Metabolic Inhibitor Alone

We examined the effects of venetoclax alone and in combination with either 2DG or 6AN on the proliferation of AML cells derived from 16 patients; 8 patients were randomly selected among the patient cluster that showed a generally strong anti-proliferative response towards treatment with metabolic inhibitors (see Figure 3B, upper cluster including 29 patients), whereas the other eight patients were selected among the lower cluster with weaker antiproliferative effects (Figure 3B). First, venetoclax was tested alone using a wide range of concentrations based on the results from a previous study [36]. The antiproliferative effects of venetoclax showed a considerable variation between patients, with no differences found between the two patient clusters (identified in Figure 3B) but a concentration-dependent antiproliferative effect was observed for most patients (Figure S15). Based on these results, 50 nM venetoclax was chosen for further studies, in combination with either 0.3 mM 2DG or 50 µM 6AN.

**Figure 8:**
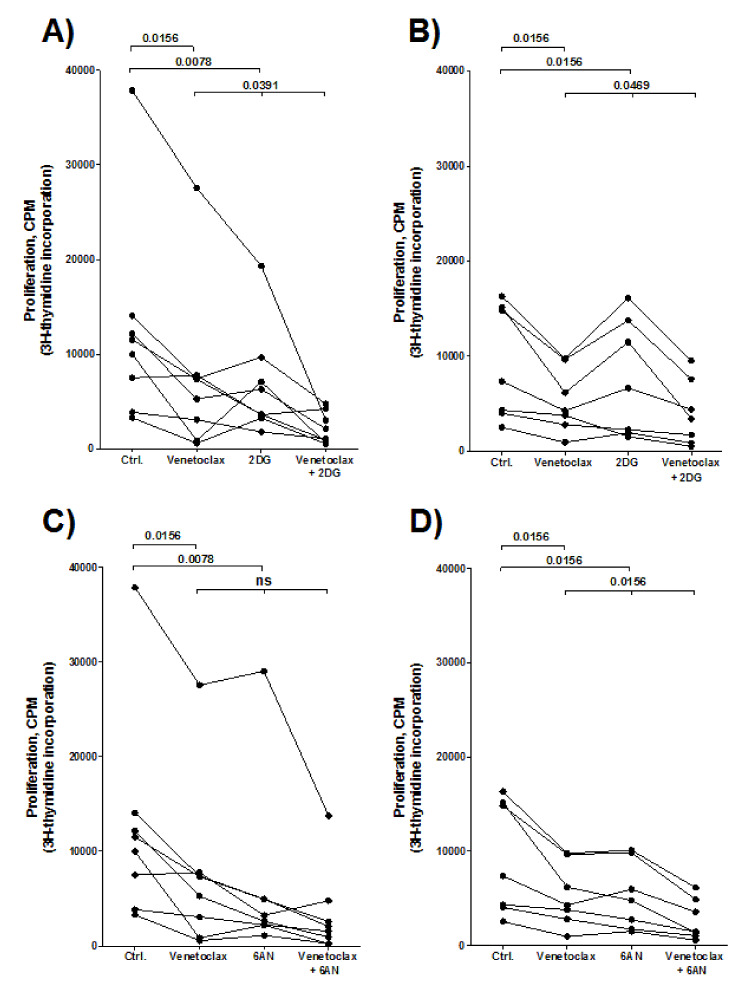
The effect of combining venetoclax with the metabolic inhibitors 2DG or 6AN on primary AML cell proliferation. The [^3^H]-thymidine incorporation assay was used to evaluate proliferation of primary AML cells after 7-day treatment of 50 nM venetoclax combined with either 0.3 mM 2DG (**A**,**B**) or 50 µM 6AN (**C**,**D**). Each graph presents the cellular proliferation shown as counts per minute (cpm) for each patient sample, that is in untreated control (Ctrl.) cultures, cultures treated with venetoclax alone, cultures treated with either 2DG or 6AN alone or combination of venetoclax with 2DG or 6AN. Samples derived from sixteen AML patients were randomly chosen among the identification of two patient clusters, identified in Figure 3B, showing different sensitivity toward metabolic inhibitors, that is eight samples (shown in **A**,**C**) from the cluster with a generally strong antiproliferative effect towards metabolic inhibitors (the 29 patients in the upper cluster), while eight other samples (shown in **B**,**D**) had a more divergent/weaker antiproliferative effect towards metabolic inhibitors (lower cluster, Figure 3B). One patient sample in our analysis showed undetectable proliferation (< 1000 cpm) and was excluded from the analysis. The *p*-values (Wilcoxon signed rank test) are shown for the antiproliferative effects of the metabolic inhibitors combined with venetoclax compared with effects observed for treatment with either the metabolic inhibitor or venetoclax alone, depending on which treatment resulted in the strongest antiproliferative effect.

For each of the sixteen patient samples, the antiproliferative effect of venetoclax combined with either 2DG or 6AN was compared with effects of treatment with either venetoclax or 2DG/6AN alone depending on the agent that showed the strongest antiproliferative effect alone (Figure 8). When comparing the overall results for each of the two patient subsets (identified in our cluster analysis, Figure 3B) we found that the antiproliferative effect of the combination of venetoclax with 2DG was significantly stronger than either of the two agents tested separately (Figure 8, *p* = 0.039 and 0.047, respectively). Also, for most patients, we found that combination of venetoclax with 6AN had an antiproliferative effect on AML cells that was stronger than for venetoclax or 6AN tested alone. The percentage inhibition of AML cell proliferation after treatment with venetoclax combined with 2DG or 6AN, did not differ between the two patient clusters (data not shown).

## 4. Discussion

Metabolic pathways are dysregulated in different types of cancer, including AML, to meet the energetic and biosynthetic demands of cancer cells. A recent study described a prognostic scoring system for cytogenetically normal AML based on the systemic levels of a limited number of metabolites, suggesting that a glucose signature had prognostic value [12]. Furthermore, in another study, glycolytic metabolism at diagnosis was associated with favorable clinical outcome [37]. It seems that AML patients are heterogeneous with regard to systemic metabolic regulation, which may reflect the metabolic characteristics of the AML cells, but also secondary or distant effects of this aggressive disease on neighboring non-leukemic cells in their common bone marrow microenvironment or effects on systemic metabolic regulation by distant organs (i.e., liver, muscles, endocrine organs) may influence the AML cells. Targeting of metabolism may be a promising therapeutic strategy in AML [4] and in this study, we have therefore investigated the direct in vitro effects of several metabolic inhibitors targeting main metabolic pathways, on the survival and proliferation of primary human AML cells. Our results from this study show that patients are heterogeneous with regard to direct effects of metabolic inhibitors on the AML cells.

We examined primary human AML cells from patients with relative and/or absolute high levels of circulating AML cells, obtaining highly enriched AML cell populations (>90%) after density gradient separation alone, with a minimal risk of inducing functional alterations as described for more extensive separation procedures [38,39]. Furthermore, our patients are representative for AML in general with regard to karyotypic abnormalities and molecular genetics [21,26,31].

Our methods for cryopreservation and thawing are highly standardized but the cells have decreased viability after thawing and will undergo spontaneous or stress-induced in vitro apoptosis during further culture [25]. However, most patient samples will have a viability of approximately 60%–70% after thawing and may show reduced expression of certain cell surface molecules [40]. Our model to analyze effects of metabolic inhibitors on AML cell viability is thus based on a combined effect of stress-induced and inhibitor-induced apoptosis. Patient samples with low stress-induced apoptosis (i.e., high viability after thawing in control cultures) also had a weak anti-viability/proapoptotic effect induced by metabolic inhibitors. Finally, for most inhibitors there was no significant association between the proapoptotic and antiproliferative effects, suggesting that the different assays may evaluate effects on different cell subsets within the hierarchically organized AML cell population, that have different characteristics.

We used the [^3^H]-thymidine incorporation assay to evaluate cell proliferation after treatment with metabolic inhibitors [41]. [^3^H]-thymidine is then added after six days of culture and nuclear incorporation is assayed one day later, that is we investigated the AML cell subset that is able to survive and still proliferate after six/seven days of culture. We selected the concentrations in our studies based on (i) concentration-response curves, (ii) no effect of the DMSO concentration (≤ 0.2%) used for dissolving inhibitors; (iii) previous experimental studies and (iv) clinical tolerated levels for those inhibitors that have so far been used in patient treatment. Metformin, 2DG, 6AN, BPTES and ST1326 all showed significant antiproliferative and proapoptotic effects, while lonidamine and AZD3965 only showed effects for a minority of patients (Figure 1 and Figure 4).

Patients with cytogenetically normal AML cells (median age 47 years, range 15-89 years) have been reported to have a distinct glucose metabolism [11] and the glycolytic profile seems important for therapy response, as well as having an impact on the clinical prognosis [37]. Glycolytic activity has been reported to be involved in the progression of several cancer types and has also been shown to contribute to chemoresistance in patients with AML [42,43]. Metformin, 2DG and 6AN are all inhibitors that have effects on glucose metabolism. 2DG and 6AN directly inhibit glucose metabolism through inhibition of glycolysis and the pentose phosphate pathway, while the main effect of metformin on glucose metabolism is through its activation of AMPK and through inhibition of OXPHOS. In addition, metformin also has a direct inhibitory effect on hexokinases, thereby inhibiting glycolysis [44,45] and its proapoptotic effect seems to depend on the glucose levels [46]. Thus, it seems that the antileukemic effects of all these three inhibitors are affected by both extracellular and intracellular glucose levels and will be most efficient on glucose dependent (glycolysis-driven) leukemia cells [46,47]. We therefore did an unsupervised hierarchical cluster analysis based on the effect of metformin, 2DG and 6AN (Figure 3A). This identified a subset of 22 patients with strong antiproliferative effects of all three inhibitors. However, we found no association between this subpopulation and any of the well-established risk factors for chemoresistance after conventional intensive chemotherapy (i.e., secondary AML, karyotype, *FLT3*-ITD, *NPM1* mutations, CD34 expression) or long-term AML-free survival.

Metabolic plasticity allows cancer cells, including leukemia cells, to reprogram their metabolism to survive during changing environments. This highly diverse and flexible metabolism of AML cells most likely not only contributes to the aggressiveness of the disease and the high percentage of refractory disease, but it can also make metabolic targeting challenging since blocking one metabolic pathway can simply make the cells shift to use another pathway to gain energy. Simultaneously targeting multiple metabolic pathways may therefore be an approach to remove their ability to survive under stressful conditions and render them vulnerable to anti-cancer treatment. Our hierarchical cluster analyses identified a patient subset with high susceptibility to five metabolic inhibitors with overall significant effects (Figure 3B, upper cluster of 29 patients). This effect was independent of the proliferative capacity of the AML cells, the spontaneous or stress-induced in vitro apoptosis and the capacity of constitutive mediator release by the AML cells and showed no association with any of the well-established risk factors for chemoresistance after conventional intensive chemotherapy or AML-free survival. Thus, the susceptibility to the antiproliferative effects of metabolic inhibitors at least partly depend on other cellular/molecular mechanisms than the susceptibility to conventional chemotherapy; this is supported by our findings of enhanced antiproliferative effects of metabolic inhibitors when tested in combination with AraC. Synergistic effects of inhibitors that target glycolytic activity in combination with chemotherapeutics have been considered as a potential therapeutic strategy in AML [48] and our data supports the further exploration of this treatment strategy.

The two patient subsets identified in our hierarchical cluster analysis based upon the antiproliferative effects after treatment with five metabolic inhibitors (Figure 3B), did not differ with regard to established biological and clinical characteristics associated with survival after intensive chemotherapy. We therefore tried to identify other AML cell characteristics associated with the antiproliferative effects of metabolic inhibitors. First, our proteomic comparisons suggest that the two patient subsets differ in metabolic regulation and also in transcriptional regulation and possibly intracellular trafficking. We would emphasize that these proteomic results should be interpreted with great care because few patients were available for these studies. Second, our comparison of global gene expression profiles showed that the two patient subsets could be identified based on mRNA profiling and these observations support the proteomic data and suggest that susceptibility to metabolic inhibitors is associated with differences in metabolic and transcriptional regulation.

Treatment with metabolic inhibitors decreased AML cell viability and had proapoptotic effects and we could identify a subset of patients with increased susceptibility to the inhibitors (Figure 5B, upper 24 patients). These patients did not differ from the others with respect to the well-established prognostic factors for patients receiving conventional intensive chemotherapy, which was similar to the findings for patients showing antiproliferative effects towards metabolic inhibitors. Thus, our viability studies support the hypothesis that the antileukemic effects of metabolic inhibitors depend on other or additional cellular mechanisms than the effects of conventional chemotherapy. Furthermore, the observed antiproliferative effects are probably not due to effects on the constitutive release of growth factors by the AML cells neither, because the effects of the inhibitors on the constitutive mediator release were divergent and relatively small compared with the persisting wide variation between individual patients.

MSCs have a cytokine-mediated supporting effect on AML cell growth and survival during in vitro culture [24] and MSCs also localize to the bone marrow in stem cell niches [49]. 2DG had a very strong antiproliferative effect on leukemic cells for a majority of AML patients and the antiproliferative effect could be maintained even in the presence of the AML-supporting MSCs. In addition, 2DG also inhibited proliferation of cocultured normal MSCs, which was most likely mediated by a direct effect on the MSCs and not an indirect effect mediated by altered mediator release by AML cells, as 2DG had relatively weak effects on the constitutive cytokine release and the wide variation between patients persisted in the presence of 2DG (Figure 7).

Recent studies suggest that AML cells have considerably metabolic plasticity [9] and our present study shows that AML cells derived from different patients are also characterized by metabolic heterogeneity, that is the antiproliferative and proapoptotic effects of various metabolic inhibitors varied between patients. This was seen after treatment with inhibitors of glycolysis but also after targeting of mitochondrial metabolism through inhibition of glutaminolysis and fatty acid oxidation. Both glutaminolysis and fatty acid oxidation are closely linked to the tricarboxylic acid cycle (TCA) cycle and thereby to oxidative phosphorylation [9] and our results thus suggest that patients are heterogeneous with regard to susceptibility toward agents targeting various metabolic pathways.

Venetoclax, a Bcl-2 inhibitor, is now used especially in the treatment of human AML for elderly and unfit patients in combination with either a hypomethylating agent or low-dose cytarabine [50,51,52]. A recent study by Pollyea et al. described that the combination of venetoclax with azacytidine modulated AML cell metabolism [53], in particular inhibiting oxidative phosphorylation. We therefore investigated the effect of combining venetoclax with the glycolytic inhibitors 2DG and 6AN. Our results showed that the combination of venetoclax with a metabolic inhibitor had a stronger antiproliferative effect than the effect of either of the agents tested alone. Taken together, based on previous studies and our present results, we suggest that combined targeting of Bcl-2 and cellular metabolism should be further explored in human AML.

Several previous studies have shown that metabolic targeting of AML cells can increase their sensitivity against other antileukemic agents. Firstly, targeting of hexokinase can result in increased sensitivity toward conventional antileukemic treatment, including both cytarabine and anthracyclines [11,54]. Glucose transporter expression can decrease the sensitivity of leukemia cells toward chemotherapy [43] and inhibition of the fructose transporter Glu5 will increase the sensitivity to cytarabine [54]. Secondly, the sensitivity toward FLT3 inhibitors can be increased by inhibition of hexokinase or glutaminase [55,56]. Finally, both amino acid metabolism (including glutaminolysis) and fatty acid oxidation seem to contribute to the sensitivity toward venetoclax [53,56,57,58,59], possibly through their effects on oxidative phosphorylation [53,57]. Thus, metabolic targeting can increase the sensitivity toward antileukemic agents, both for conventional chemotherapy and targeted therapies. Our present studies suggest that such sensitizing effects, at least for cytarabine, will differ between patients.

The metabolic status of AML cells can also be influenced by the amount of nutrients and metabolites in their microenvironment [9,10,46,60]. Modulation of systemic metabolic profiles may therefore be a strategy that indirectly affects the AML cells through alteration of their microenvironment. Possible strategies could then be treatment with metformin and statins [60,61]. However, another alternative could be treatment with valproic acid that alters metabolic profiles, especially levels of several lipids/fatty acids and amino acid metabolites [62]. Thus, combined direct and indirect metabolic targeting of the AML cells may be an alternative treatment option.

Some of the metabolic inhibitors in our present studies could also reduce the proliferation of normal UCB cells, and 2DG also decreased the proliferation of normal bone marrow MSCs. Especially the risk of hematological toxicity will be important when using metabolic inhibitors in AML therapy, because these patients have leukemia-induced bone marrow failure and additional treatment-induced hematological toxicity is common. Five of the agents in our study have been or are included in ongoing registered clinical trials according to the ClinicalTrials or PubMed databases, though, as far as we know, only clinical trials with metformin have included AML patients. With regard to side effects, the most common side effects of metformin are metabolic and gastrointestinal toxicity [63,64], which is also most common for 2DG along with cardiologic side effects [65]. For 6AN, gastrointestinal side effects and toxicity to the eight cranial nerve are most common [66]. The agents BPTES and ST1326 are not registered for clinical trials, however other agents directed against similar molecular targets (glutaminolysis and fatty acid oxidation) are included in clinical trials against cancer. Hepatotoxicity was most common for the GSL1 inhibitor CB-839 [66] and the CPT-1 inhibitor etomoxir [67]. Lonidamine seems to be well tolerated [68,69] and the dose-limiting toxicity of AZD3965 is retinal side effects [70]. Finally, fatigue and dizziness have been reported as side effects for some of the metabolic inhibitors. Thus, despite our findings showing inhibitory effects of agents targeting metabolism on UCB cells and bone marrow MSCs, severe hematological toxicity has not been reported in any of these studies. In addition, the antiproliferative effect may be stronger on AML cells than for at least certain normal cells and for the UCB cells we detected only antiproliferative effects but not additional decreased cell viability as we observed for the leukemic cells. Our findings with MSCs and UCB cells thus support the observations suggested by clinical studies including patients with other disorders than AML, that the toxicity of metabolic inhibitors seems to be acceptable. In our opinion, metabolic targeting should therefore be further investigated in AML, but our present results suggest that careful evaluation for early detection of especially hematological toxicity will be important.

## 5. Conclusions

We investigated the effects of seven metabolic inhibitors on AML cells derived from 81 patients, which target various metabolic pathways including glycolysis, the pentose phosphate pathway, glutaminolysis and fatty acid oxidation. Several of these inhibitors also indirectly target OXPHOS. The metabolic inhibitors had antiproliferative and proapoptotic effects on leukemia cells for most AML patients. The inhibitor with the strongest effect varied between individual patients, although inhibition of glycolysis had the strongest effect for the majority of patients. Our overall results suggest that several strategies for metabolic inhibition have antileukemic effects, but the treatment should possibly be individualized because the optimal inhibitor seems to vary between patients. Thus, the various strategies for metabolic targeting in AML should be further characterized, especially in patient-derived xenograft models, but also in clinical studies and there should be a focus on patient heterogeneity in these studies. Further studies should also evaluate effects on the leukemic stem cell population that are thought to contribute to relapsed/refractory disease. In our opinion, the clinical studies should include both AML stabilizing studies and combination with chemotherapy and potentially curative treatments.

## Figures and Tables

**Table 1:** Biological and clinical features of the 81 AML patients in this study.

CLINICAL CHARACTERISTICS					
*Gender (n)*			*Age (years)*		
Female Male		30 51	Median Range		67.5 17–87
*Relapse or previous hematological disease*			Survival*		
AML relapse MDS CMML PV Li-Fraumeni syndrome		5 11 2 1 1	Yes No Not relevant		15 24 42
**AML CELL DIFFERENTIATION**					
*FAB classification*			*CD34 expression*		
M0/M1/M2 M4/M5 nd		38 36 7	Negative (<20%) Positive (≥20%) nd		22 52 7
**GENETIC ABNORMALITIES**					
Cytogenetics**		*FLT3*		*NPM-1*	
Favorable Intermediate Adverse nd	10 48 14 9	wt ITD TKD ITD/TKD nd	45 22 2 3 9	wt INS nd	45 28 8

Abbreviations: CMML, chronic myelomonocytic leukemia; FAB, French-American-British; INS, insertion; ITD, internal tandem duplication; MDS, myelodysplastic syndrome; nd, not determined; PV, polycythemia vera; TKD, tyrosine kinase domain; wt, wild type. *Patients completing potentially curative intensive induction and consolidation therapy and being alive or dead from relapse after at least two years of follow-up. Not relevant means that the patients received only AML-stabilizing treatment or did not complete planned intensive therapy. **The European LeukemiaNet (ELN-2017) classification was used.

**Table 2:** Main targets and metabolic effects of the seven metabolic inhibitors tested on AML cells in this study. (For more detailed information and references, see Table S1).

Metabolic Inhibitor	Molecular Target—Main Metabolic Pathway Affected
AZD3965	Inhibits *glycolysis*; a selective inhibitor of monocarboxylate transporter 1 (MCT1) that regulates lactate transport across the plasma membrane.
Metformin	Inhibits *oxidative phosphorylation* (OXPHOS) and *fatty acid metabolism;* inhibits hexokinase activity, activates AMP-activated protein kinase (AMPK) and indirectly inhibits mammalian target of rapamycin (mTOR).
2DG	Inhibits *glycolysis*; inhibits the rate-limiting enzyme hexokinase.
Lonidamine	Inhibits *glycolysis and OXPHOS* through multisite effects, including inhibition of hexokinase II, MCT1, the mitochondrial pyruvate carrier, the electron transport chain and alters mitochondrial permeability.
6AN	Inhibits the *pentose phosphate pathway* (PPP) which is a main source of NADPH and ribose-5 phosphate.
BPTES	Inhibits *glutaminase* activity, that is the conversion of glutamine to glutamate.
ST1326	Inhibits carnetyl palmitoyl transferase-1 (CPT-1), the rate-limiting step of *fatty acid oxidation* (FAO).

## Supplementary Materials

The following are available online at https://www.mdpi.com/2073-4409/9/5/1155/s1, Figure S1: Initial dose-response studies of seven metabolic inhibitors; effects on the proliferation of primary human AML cells and umbilical cord blood (UCB) mononuclear cells, Figure S2: Effects of treatment with metabolic inhibitors or AraC on AML cell proliferation, Figure S3: Effects of 2DG treatment on primary AML cell proliferation and viability, Figure S4: Effects of treatment with the glycolytic inhibitor 2DG or AraC on AML cell proliferation, Figure S5: AML associated molecular genetics for 35 unselected patients (i.e., a consecutive subset from our patient cohort), a summary of the overall results, Figure S6: Hierarchical cluster analysis based on AML cell proliferation in cultures treated with and without metabolic inhibitors, Figure S7: A comparison of global gene expression profiles for AML samples showing a strong antiproliferative effect towards metabolic inhibitors versus no or minimal antiproliferative effect (based on Figure 3B, upper and lower cluster, respectively), Figure S8: Effects of metabolic inhibitors on AML cell viability and apoptosis, Figure S9: Flow cytometric analysis of AML cell viability; fraction of viable, apoptotic and late apoptotic/necrotic AML cells in cultures treated with or without metabolic inhibitors, Figure S10: Antiproliferative and proapoptotic effects of metabolic inhibitors on primary human AML cells, Figure S11: Unsupervised hierarchical cluster analysis of the constitutive release of soluble mediators by primary AML cells derived from 72 unselected patients, that is the patients with >5% viable cells after 48 h of in vitro culture, Figure S12: The constitutive release of MMP2 and MMP9 by primary human AML cells derived from 78 patients; effects of metformin, 2DG or 6AN, Figure S13: Effects of 2DG on supernatant mediator levels after transwell coculture of primary human AML cells and normal MSCs, Figure S14: Viability of UCB cells derived from five donors after 48-hour treatment with metabolic inhibitors compared to untreated controls, Figure S15: Effect of venetoclax on AML cell proliferation; a comparison of two patient clusters with different sensitivity towards metabolic inhibitors (Figure 3B). Table S1: Main targets of the seven metabolic inhibitors tested on AML cells, Table S2: A comparison of clinical/biological characteristics of AML patients when comparing patient samples with antiproliferative effects greater or less than 20% inhibition after treatment with metabolic inhibitors, Table S3: A comparison of the two patient subsets identified in the unsupervised hierarchical cluster analysis based on the antiproliferative effects of five metabolic inhibitors (Figure 3B), Table S4: Proteomic comparison of primary AML cells derived from patients where AML cell proliferation is either strongly inhibited by metabolic inhibitors (6 patients) or AML cells show no or minimal antiproliferative effects by metabolic inhibitors (8 patients) (based on Figure 3B), Table S5: Description of the 31 differentially expressed proteins found after performing a proteomic comparison between a subset of patient samples showing a strong antiproliferative effect towards metabolic inhibitors (n=6) versus patient samples that show no or low effect towards metabolic inhibitors (n=8) (Figure 3B), Table S6: Functional network analysis based on a proteomic comparison of a subset of patients showing a strong antiproliferative effect towards metabolic inhibitors versus patients that show no or low antiproliferative effects towards metabolic inhibitors in vitro (Figure 3B), Table S7: A comparison of the two patient subsets identified in the unsupervised hierarchical cluster analysis based on AML cell viability after treatment with metabolic inhibitors (Figure 5B).
