# Supplementary material for: Targeting Cellular Metabolism in Acute Myeloid Leukemia and the Role of Patient Heterogeneity
Source: Cells. 2020 May 7;9(5):1155. doi: 10.3390/cells9051155 (PMC7290417; doi:10.3390/cells9051155)
Supplement: Supplementary file 1 [file cells-09-01155-s001.pdf]

SUPPLEMENTARY INFORMATION

TARGETING CELLULAR METABOLISM IN ACUTE MYELOID LEUKEMIA AND THE ROLE OF PATIENT HETEROGENEITY

Ida-Sofie Grønningsæter[1,2], Håkon Reikvam[1,2], Elise Aasebø[1], Sushma Bartaula-Brevik[1], Tor Henrik Tvedt[1,2], Øystein Bruserud[1,2]* and Kimberley Joanne Hatfield[1,3]*

[1]Department of Clinical Science, University of Bergen, Bergen, Norway, [2]Department of Medicine, Haukeland University Hospital, Bergen, Norway and [3]Department of Immunology and Transfusion Medicine, Haukeland University Hospital, Bergen, Norway.

* Correspondence: Kimberley.Hatfield@uib.no, Tel.: +47 55973037; Oystein.bruserud@helse-bergen.no, Tel.: +47-55973082; Fax +47-55972950

Ida.Gronningseter@uib.no

Hakon.Reikvam@uib.no

Elise Aasebø Elise.Aasebo@uib.no

Sushma Bartaula-Brevik <Sushma.Bartaula@uib.no>

tor.henrik.anderson.tvedt@helse-bergen.no

Oystein.bruserud@helse-bergen.no

Kimberley.Hatfield@uib.no

Table S1. Main targets of the seven metabolic inhibitors tested on AML cells. The table presents the inhibitors used in this study and their main molecular targets, and a brief description of the biological effects for each inhibitor.

**Metabolic inhibitor**	**Molecular target –** ***metabolic pathway***	**Biological effects**
AZD3965	MCT1 – *Glycolysis (Lactate/Pyruvate)*	AZD3965 is a second generation selective MCT1 inhibitor. It inhibits both lactate influx and efflux into cells obstructing the metabolic interplay between cell populations within tumors. In addition, it increases glycolysis and upregulates glycolytic enzymes [1, 2].
Metformin	*OXPHOS and AMPK signaling pathway*	Metformin inhibits mTOR indirectly through activation of LKB1/AMPK. It inhibits OXPHOS and fatty acid metabolism, which in turn increases glycolysis [3-5].
2DG	Hexokinase – *Glycolysis*	2DG is a glucose analogue that inhibits hexokinase, the first and rate-limiting enzyme in glycolysis [6, 7].
Lonidamine	Hexokinase II – *Glycolysis*<br>*OXPHOS*	Lonidamine has a multisite effect. It targets hexokinase II, and also the mitochondrial pyruvate carrier, mitochondrial permeability, MCT and the electron transport chain [8, 9].
6AN	G6PD and 6PGD - *PPP*	6AN is a nicotinamide analogue and a competitive inhibitor of PPP, a parallel metabolic pathway to glycolysis. PPP directs glucose to its oxidative branch leading to production of NADPH, but it is also a key autophagy-dependent compensatory metabolic pathway [10, 11].
BPTES	Glutaminase - *Glutaminolysis*	It inhibits glutaminase, the enzyme that converts glutamine to glutamate which is further oxidized to α-ketoglutarate. Glutamine is an important source of energy for many cancer cells. This is especially seen for *IDH* mutated cells. Glutaminase is upregulated by the *MYC* oncogene [12-14].
ST1326	CPT1 – *Fatty acid metabolism*	FAO is an important source of fuel for cancer cells` proliferation during metabolic stress. CPT1 catalyzes the rate-limiting step of FAO, and ST1326 is a selective CPT1 inhibitor [15].

Abbreviations: 6AN, 6-Aminonicotinamide; AMPK, AMP-activated protein kinase; CPT-1, carnetyl palmitoyl transferase-1; 2DG, 2-Deoxy-D-glucose; FAO, fatty acid oxidation; G6PD, glucose-6-phosphate dehydrogenase; LKB1, liver kinase B1; MCT1, monocarboxylate transporter 1; OXPHOS, oxidative phosphorylation; 6PGD, 6-phosphogluconate dehydrogenase; PPP, pentose phosphate pathway.

Table S2. A comparison of clinical/biological characteristics of AML patients when comparing patient samples with antiproliferative effects greater or less than 20% inhibition after treatment with metabolic inhibitors. The comparisons (secondary vs. *de novo*, FAB; M0-M2 vs. M4/M5, cytogenetics; favorable, intermediate and adverse, CD34; positive vs. negative, *FLT3* mutation (ITD) vs. wt, *NPM1* mutation (INS) vs. wt, and survivors vs. nonsurvivors) are based on the percent inhibition (< or > 20% inhibitory effect) of metabolic inhibitors on cell proliferation in treated cultures compared to untreated control cultures, for each inhibitor and patient (i.e. 69 patients showing detectable proliferation in control cultures; 53 patients for ST1326). Differences were regarded as statistically significant when $p < 0.05$ (shown in bold). The Mann-Whitney *U-test* and the Kruskal-Wallis test were used for the statistical analyses, and the *p*-values are not corrected for the number of comparisons.

Metabolic inhibitors and concentrations	Secondary versus *de novo*	FAB	Cytogenetics	CD34 expression	*FLT3* (ITD versus wt)	*NPM1* (INS versus wt)	Survivors versus nonsurvivors
Metformin 2.5 mM	0.549	0.071	0.190	0.114	0.903	0.385	0.736
Metformin 1.25 mM	0.303	0.205	0.303	0.073	0.942	0.198	0.911
2DG 0.6 mM	0.242	0.065	0.074	0.207	0.261	0.472	0.881
2DG 0.3 mM	0.578	0.508	**0.032**	0.438	0.680	0.403	0.600
6AN 100 µM	0.075	0.910	0.098	0.605	0.512	0854	0.295
6AN 50 µM	**0.010**	0.422	**0.045**	0.879	0.104	0.815	0.575
BPTES 20 µM	0.956	0.367	0.889	0.117	0.633	0.462	0.501
BPTES 10 µM	0.989	0.662	0.971	0.124	0.599	0.341	0.575
ST1326 50 µM	0.790	0.590	0.899	0.431	0.015	0.103	0.910
Lonidamine 300 µM	0.606	0.833	0.389	0.148	0.512	0.155	1
AZD3965 200 nM	0.824	0.135	0.421	0.162	0.544	0.483	0.765

Table S3. A comparison of the two patient subsets identified in the unsupervised hierarchical cluster analysis based on the antiproliferative effects of five metabolic inhibitors (Figure 3B). The anti-proliferative effect induced by each metabolic inhibitor was calculated for each of the 69 patients (53 patients for ST1326) showing detectable cell proliferation in the [^{3}H]-thymidine incorporation assay (i.e. 7-day suspension cultures) (Figure 1). A hierarchical cluster analysis was done based on the percent inhibition by metabolic inhibitors, and we then identified two clusters (Figure 3B), where the upper cluster included 29 patients that showed a strong antiproliferative effect towards metabolic inhibitors. The table shows (from left to right); the inhibitors tested, the cluster identified (weak or strong effect towards metabolic inhibitors), the median proliferation (percentage of proliferation in inhibitor-treated cultures relative to corresponding control cultures) and the *p*-value (Mann-Whitney *U-test*). Differences were regarded as statistically significant when $p \leq 0.05$ (no corrections were made for the number of comparisons, significant *p*-values marked in bold).

Metabolic inhibitor	Cluster (i.e. anti-proliferative effect)	Number of patients in each group	Median proliferation (%) of inhibitor-treated cultures relative to control cultures	*p*-value
Metformin 2.5 mM	Weak effect	40	64.0	
	Strong effect	29	48.6	**0.024**
2DG 0.6 mM	Weak effect	40	61.6	
	Strong effect	29	12.3	**0.000**
6AN 100 µM	Weak effect	40	78.9	
	Strong effect	29	26.5	**0.000**
BPTES 20 µM	Weak effect	40	70.9	
	Strong effect	29	47.2	**0.003**
ST1326 50 µM	Weak effect	31	52.4	
	Strong effect	22	27.7	0.097

Table S4. Proteomic comparison of primary AML cells derived from patients where AML cell proliferation is either strongly inhibited by metabolic inhibitors (6 patients) or AML cells show no or minimal antiproliferative effects by metabolic inhibitors (8 patients) (based on Figure 3B). The analysis identified 31 differentially expressed proteins, and the table shows a functional classification of the individual proteins (for a detailed description see Table S5). Some of the proteins are included in more than one functional class.

**Functional classification**	**INCREASED in subset susceptible to metabolic inhibitors (18 proteins)**	**DECREASED in subset susceptible to metabolic inhibitors (13 proteins)**
**Metabolism (n=15)**		
Protein/proteinases/molecular degradation/amino acid	PRTN3, CFD, THEM6, HM13,	UBXN7, QRICH1, DDAH2, ISOC1
Lipid	SGPL1, AGPS	
Energy	FDXR	DDAH2, ISOC1, ACO1
Detoxification	SQRDL	
**Mitochondria (n=4)**	SQRDL, FDXR	DDAH2, ACO1
**Transcription, RNA (n=9)**	RNASE3, RNASE 2, SRPK1, ASCC3	RFX1;RFX2, QRICH1, SMNDC1, GRWD1, ACO1
**DNA damage (n=2)**	ASCC3	GRWD1
**Cell cycle regulation, cytoskeleton (n=3)**		NAE1, MAPRE2, MPP1
**Intracellular signaling (n=8)**	BST1, IL1RAP	PIP4K2A, MPP1
G protein coupled receptor	SGPL1, CD97	RAPIGAP2
Kinase/phosphatase	SRPK1	
**Intracellular transport, Golgi, endoplasmic reticulum, microtubule (n=3)**	TMEM214, HM13,	MAPRE2
**Cell membrane, adhesion, migration (n=6)**	BST1, HLA-B, CD97, IL1RAP, CPNE2,	MPP1
**Apoptosis (n=1)**	TMEM214	
**Cancer association (n=7)**	BST1, CD97	RFX1, MAPRE2, GRWD1, MPP1, ISOC1

Table S5. Description of the 31 differentially expressed proteins found after performing a proteomic comparison between a subset of patient samples showing a strong antiproliferative effect towards metabolic inhibitors (n=6) versus patient samples that show no or low effect towards metabolic inhibitors (n=8) (Figure 3B). Table is based on information from https://www.ncbi.nlm.nih.gov/gene/.

Gene name	Protein name	Key words
**INCREASED (18 proteins)**		
PRTN3	*Myeloblastin/proteinase 3.* Restricted expression to bone marrow. This is an AML-associated antigen.	Proteinase
RNASE3	*Eosinophil cationic protein/ ribonuclease A family member 3.* The protein belongs to the pancreatic ribonuclease family, a subset of the ribonuclease A superfamily. Its expression is regulated by GATA transcription factors. Restricted expression to bone marrow.	RNAse
CFD	*Complement factor D.* This gene encodes a member of the S1, or chymotrypsin, family of serine peptidases. This protease catalyzes the cleavage of factor B, the rate-limiting step of the alternative pathway of complement activation. This protein also functions as an adipokine, a cell signaling protein secreted by adipocytes, which regulates insulin secretion.	Serine proteinase<br>Insulin secretion
RNASE2	*Non-secretory ribonuclease/ ribonuclease A family member 2.* The protein is a non-secretory ribonuclease that belongs to the pancreatic ribonuclease family, a subset of the ribonuclease A superfamily. Restricted expression to bone marrow.	RNAse
BST1	*ADP-ribosyl cyclase/cyclic ADP-ribose hydrolase 2/ bone marrow stromal cell antigen 1.* This is a glycosylphosphatidylinositol-anchored molecule with high expression in normal bone marrow. CD157/Bst1 is a dual-function receptor and β-NAD$^+$-metabolizing ectoenzyme of the ADP-ribosyl cyclase family. CD157 interacts with extracellular matrix components and regulates leukocyte diapedesis via integrin-mediated signaling; it also regulates cell migration and is a marker of adverse prognosis in certain cancers.	Metabolism<br>Cell adhesion<br>Signaling<br>Cancer
HLA-B	*HLA class I histocompatibility antigen, B alpha chain.* Belongs to the HLA class I heavy chain paralogues. This class I molecule is a heterodimer consisting of a heavy chain and a light chain (beta-2 microglobulin). Class I molecules play a central role in the immune system by presenting peptides derived from the endoplasmic reticulum lumen. They are expressed in nearly all cells.	Cell surface<br>HLA
SGPL1	*Sphingosine-1-phosphate lyase 1.* Low, but detectable expression in normal bone marrow. *SGPL1* is the intracellular enzyme responsible for the irreversible final breakdown of the lipid molecule S1P, which is cleaved to ethanolamine phosphate and *trans*-2-hexadecenal. Through G-protein-coupled receptor activation, it has been proven that S1P has important regulatory functions in normal physiology. The molecule is localized in the endoplasmic reticulum.	Lipid metabolism<br>G-protein coupled receptor
SQRDL	*Sulfide Quinone oxidoreductase, mitochondrial.* The protein may function in mitochondria to catalyze the conversion of sulfide to persulfides, thereby decreasing toxic concentrations of sulfide.	Mitochondria<br>Sulfide detoxification
CD97	*CD97 antigen/adhesion G protein-coupled receptor E5.* This protein is a member of the EGF-TM7 subfamily of adhesion G protein-coupled receptors, which mediate cell-cell interactions. These proteins are cleaved by self-catalytic proteolysis into a large extracellular subunit and seven-span transmembrane subunit, which associate at the cell surface as a receptor complex. The encoded protein may play a role in cell adhesion as well as leukocyte recruitment, activation and migration, and contains multiple extracellular EGF-like repeats, which mediate binding to chondroitin sulfate and the cell surface complement regulatory protein CD55. Its expression may play a role in the progression of several types of cancer.	Cell surface<br>GPCR<br>Cell adhesion<br>Cell migration<br>Carcinogenesis

IL1RAP	*Interleukin-1 receptor accessory protein.* This protein is a component of the interleukin 1 receptor complex. Alternative splicing of this gene results in membrane-bound and soluble isoforms differing in their C-terminus. The ratio of soluble to membrane-bound forms increases during acute-phase induction or stress.	Signaling
FDXR	*NADPH adrenodoxin oxidoreductase, mitochondrial/ferredoxin reductase.* This mitochondrial flavoprotein initiates electron transport for cytochromes P450 receiving electrons from NADPH.	Mitochondria<br>Metabolism
AGPS	*Alkyldihydroxyacetonephosphate synthase, peroxisomal.* This protein is a member of the FAD-binding oxidoreductase/transferase type 4 family and catalyzes the second step of ether lipid biosynthesis in which acyl-dihydroxyacetonephosphate (DHAP) is converted to alkyl-DHAP by the addition of a long chain alcohol and the removal of a long-chain acid anion. The protein is localized to the inner aspect of the peroxisomal membrane and requires FAD as a cofactor.	Metabolism<br>Lipid
TMEM214	*Transmembrane protein 214.* Endoplasmic reticulum (ER) stress caused by excessive aggregation of misfolded proteins induces apoptosis. TMEM214 is suggested to be a critical mediator of ER stress-induced apoptosis. Overexpression of TMEM214 induced apoptosis, whereas knockdown of TMEM214 inhibited ER stress-induced apoptosis. TMEM214 can localize on the outer membrane of the ER where it is constitutively associated with procaspase 4, which is also critical for ER stress-induced apoptosis.	Apoptosis<br>Endoplasmic reticulum
SRPK1	*SRSF protein kinase 1.* This serine/arginine protein kinase specific for the SR (serine/arginine-rich domain) family of splicing factors. The protein localizes to the nucleus and the cytoplasm. It is thought to play a role in regulation of both constitutive and alternative splicing by regulating intracellular localization of splicing factors.	RNA splicing<br>Kinase
THEM6	*Protein THEM6/thioesterase superfamily member 6.* THEM6 is a protein-coding gene.	Esterase
CPNE2	*Copine-2.* Calcium-dependent membrane-binding proteins may regulate molecular events at the interface of the cell membrane and cytoplasm. This gene encodes a calcium-dependent protein containing two N-terminal type II C2 domains and an integrin A domain-like sequence in the C-terminus.	Cell membrane<br>Calcium
HM13	*Minor histocompatibility antigen H13.* The protein localizes to the endoplasmic reticulum, catalyzes intramembrane proteolysis. Its activity is required to generate signal sequence-derived HLA-E epitopes that are recognized by the immune system.	Endoplasmic reticulum<br>Proteolysis
ASCC3	*Activating signal cointegrator 1 complex subunit 3.* This protein belongs to a family of helicases that are involved in the ATP-dependent unwinding of nucleic acid duplexes. The encoded protein is the largest subunit of the activating signal cointegrator 1 complex that is involved in DNA repair and resistance to alkylation damage.	Helicase
**DECREASED (13 proteins)**		
PIP4K2A	*Phosphatidylinositol 5-phosphate 4-kinase type-2 alpha.* Phosphatidylinositol-5,4-bisphosphate, the precursor to second messengers of the phosphoinositide signal transduction pathways. The protein encoded by this gene is one of a family of enzymes capable of catalyzing the phosphorylation of phosphatidylinositol-5-phosphate This gene is a member of the phosphatidylinositol-5-phosphate 4-kinase family.	Phosphoinositide<br>Signaling
RFX1;RFX2	*MHC class II regulatory factor RFX1; DNA-binding protein RFX2.* This gene encodes a member of the regulatory factor X (RFX) family of transcription factors. RFX1 contains an N-terminal activation domain and a C-terminal repression domain, and may activate or repress target gene expression depending on cellular context. This transcription factor has been shown to regulate a wide variety	Transcription factor

	of genes involved in cancer. RFX2 is a transcriptional activator that can bind DNA as a monomer or as a heterodimer with other RFX family members.	
NAE1	*NEDD8-activating enzyme E1 regulatory subunit.* The protein binds to the beta-amyloid precursor protein, a cell surface protein with signal-transducing properties. It can form a heterodimer with UBE1C and bind and activate NEDD8, an ubiquitin-like protein. This protein is required for cell cycle progression through the S/M checkpoint.	Cell cycle<br>S/M checkpoint
MAPRE2	*Microtubule-associated protein RP/EB family member 2.* The protein shares significant homology to the adenomatous polyposis coli (APC) protein-binding EB1 gene family. It is a microtubule-associated protein that is necessary for spindle symmetry during mitosis. It is thought to play a role in control of proliferation.	Microtubule<br>Mitosis
QRICH1	*Glutamine-rich protein 1.* The molecule has a caspase activating domain, it is predicted to localize to the nucleus and may be involved in transcriptional regulation.	Protease<br>Transcription
UBXN7	*UBX domain-containing protein 7.* UBXN7 over-expression converts the scaffold protein CUL2 to its neddylated form and causes the accumulation of non-ubiquitylated HIF1α.	Transcription
SMNDC1	*Survival of motor neuron-related-splicing factor 30.* This is a nuclear protein that has been identified as a constituent of the spliceosome complex.	RNA<br>Splicing
GRWD1	*Glutamate-rich WD repeat-containing protein 1.* This protein may play a critical role in ribosome biogenesis and may also have a role in histone methylation through interactions with CUL4-DDB1 ubiquitin E3 ligase. GRWD1 negatively regulates p53 via the RPL11-MDM2 pathway and promotes tumorigenesis.	Ribosome<br>Histone<br>Transcription<br>p53
RAP1GAP2	*Rap1 GTPase-activating protein 2.* This gene encodes a GTPase-activating protein that activates the small guanine-nucleotide-binding protein Rap1. The protein interacts with synaptotagmin-like protein 1 and Rab27.	GTP-ase
MPP1	*55 kDa erythrocyte membrane protein/ membrane palmitoylated protein 1.* This gene encodes the prototype of the membrane-associated guanylate kinase (MAGUK) family proteins. MAGUKs interact with the cytoskeleton and regulate cell proliferation, signaling pathways, and intercellular junctions. The encoded protein is an extensively palmitoylated membrane phosphoprotein containing a PDZ domain, a Src homology 3 (SH3) motif, and a guanylate kinase domain. This gene product interacts with various cytoskeletal proteins and cell junctional proteins. It may be important for tumor suppressor activity.	Guanylate kinase<br>Cytoskeleton<br>Cell junctions
DDAH2	*N(G),N(G)-dimethylarginine dimethylaminohydrolase 2.* This dimethylarginine dimethylaminohydrolase functions in nitric oxide generation by regulating the cellular concentrations of methylarginines, which in turn inhibit nitric oxide synthase activity. The protein may be localized to the mitochondria.	Mitochondria<br>Nitric oxide
ISOC1	*Isochorismatase domain-containing protein 1.* This protein has putative isochorismatase activity and is a regulator of proliferation and survival in pancreatic cancer cells.	Hydralase<br>Pyruvate<br>Carcinogenesis
ACO1	*Cytoplasmic aconitate hydratase.* The protein is a multi-functional, cytosolic protein that functions as an essential enzyme in the TCA cycle and interacts with mRNA to control the levels of iron inside cells. When cellular iron levels are high, this protein binds to a 4Fe-4S cluster and functions as an aconitase. Aconitases are iron-sulfur proteins that function to catalyze the conversion of citrate to isocitrate. When cellular iron levels are low, the protein binds to iron-responsive elements (IREs), which are stem-loop structures found in the 5' UTR of ferritin mRNA, and in the 3' UTR of transferrin receptor mRNA. When the protein binds to IRE, it results in	Mitochondria<br>Metabolism<br>Transcription<br>Leukemia

	repression of translation of ferritin mRNA, and inhibition of degradation of the otherwise rapidly degraded transferrin receptor mRNA. The protein is involved in the metabolic shift during development of acute lymphoblastic leukemia.

Table S6. Functional network analysis based on a proteomic comparison of a subset of patients showing a strong antiproliferative effect towards metabolic inhibitors versus patients that show no or low antiproliferative effects towards metabolic inhibitors *in vitro* (Figure 3B). Based on 49 proteins with significant difference, 17 proteins interacted with other proteins, forming protein networks; and a description of the network-associated proteins are shown below. A black circle around the node indicates a significant fold change, while a statistically significant difference is indicated as * in the table. Description of proteins is based on information from https://www.ncbi.nlm.nih.gov/gene/.

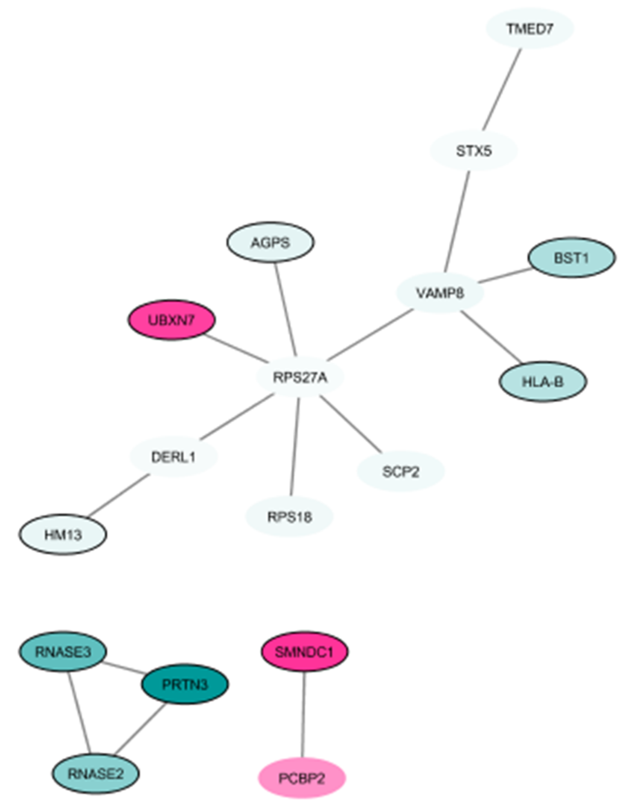

Gene name	Protein name	Key words
**INCREASED (14 proteins)**		
*PRTN3**	*Myeloblastin/proteinase 3.* Restricted expression to bone marrow. This is an AML-associated antigen.	Proteinase

*RNASE3**	*Eosinophil cationic protein/ ribonuclease A family member 3.* The protein belongs to the pancreatic ribonuclease family, a subset of the ribonuclease A superfamily. Its expression is regulated by GATA transcription factors. Restricted expression to bone marrow.	RNAse
*RNASE2**	*Non-secretory ribonuclease/ ribonuclease A family member 2.* The protein is a non-secretory ribonuclease that belongs to the pancreatic ribonuclease family, a subset of the ribonuclease A superfamily. Restricted expression to bone marrow.	RNAse
*BST1**	*ADP-ribosyl cyclase/cyclic ADP-ribose hydrolase 2/ bone marrow stromal cell antigen 1.* This is a glycosylphosphatidylinositol-anchored molecule with high expression in normal bone marrow. CD157/Bst1 is a dual-function receptor and β-NAD+-metabolizing ectoenzyme of the ADP-ribosyl cyclase family. CD157 interacts with extracellular matrix components and regulates leukocyte diapedesis via integrin-mediated signaling. CD157 also regulates cell migration and is a marker of adverse prognosis in certain cancers.	Metabolism<br>Cell adhesion<br>Signaling<br>Cancer
*HLA-B**	*HLA class I histocompatibility antigen, B alpha chain.*	Cell surface<br>HLA
*AGPS**	*Alkyldihydroxyacetonephosphate synthase, peroxisomal.* This protein is a member of the FAD-binding oxidoreductase/transferase type 4 family and catalyzes the second step of ether lipid biosynthesis in which acyl-dihydroxyacetonephosphate (DHAP) is converted to alkyl-DHAP by the addition of a long chain alcohol and the removal of a long-chain acid anion. The protein is localized to the inner aspect of the peroxisomal membrane and requires FAD as a cofactor.	Metabolism<br>Lipid
*HM13**	*Minor histocompatibility antigen H13.* The protein localizes to the endoplasmic reticulum, catalyzes intramembrane proteolysis. Its activity is required to generate signal sequence-derived HLA-E epitopes that are recognized by the immune system. The encoded protein is an integral membrane protein with sequence motifs characteristic of the presenilin-type aspartic proteases.	Endoplasmic reticulum<br>Proteolysis
TMED7	T*ransmembrane p24 trafficking protein 7.* This protein inhibits MyD88-independent toll-like receptor 4 signaling. TMED7 overexpression inhibits the ability of TRAM, an adaptor utilized by toll-like receptor (TLR) 4 to activate the interferon regulatory factor 3-signalling pathway, whereas TMED7 knockdown enhances production of the CCL5 chemokine following TLR4 ligation. Upon lipopolysaccharide stimulation, TMED7 co-localizes with TRAM and TLR4 in late endosomes where it encounters the negative regulator of TRAM, TAG. TMED7 is essential for TAG-mediated disruption of the TRAM/TRIF complex and the degradation of TLR4.	TLR4<br>CCL5<br>Myd88
STX5	*Syntaxin 5.* This gene encodes a member of the syntaxin or t-SNARE (target-SNAP receptor) family. These proteins are found on cell membranes and serve as the targets for v-SNAREs (vesicle-SNAP receptors), permitting specific synaptic vesicle docking and fusion. The encoded protein regulates endoplasmic reticulum to Golgi transport and plays a critical role in autophagy.	Endoplasmic reticulum<br>Golgi<br>Autophagy
VAMP8	*Vesicle associated membrane protein 8.* This gene encodes an integral membrane protein that belongs to the synaptobrevin/vesicle-associated membrane protein subfamily of soluble N-ethylmaleimide-sensitive factor attachment protein receptors (SNAREs). The encoded protein is involved in the fusion of synaptic vesicles with the presynaptic membrane.	Endoplasmic reticulum<br>Golgi<br>Autophagy
RPS27A	*Ribosomal protein S27a.* Ubiquitin, a highly conserved protein that has a major role in targeting cellular proteins for degradation by the 26S proteosome, is synthesized as a precursor protein consisting of either polyubiquitin chains or a single ubiquitin fused to an unrelated protein. This gene encodes a fusion protein consisting of ubiquitin at the N-terminus and ribosomal protein S27a at the C-terminus. Ribosomal protein S27a is a component of the 40S subunit of the ribosome and belongs to the S27AE family of ribosomal proteins. It contains C4-type zinc finger domains and is located in the cytoplasm.	Ribosome<br>Proteasome<br>Ubiquitin
SCP 2	SCP2 protein is thought to be an intracellular non-specific lipid transfer protein. Alternative splicing of this gene produces multiple transcript variants, some encoding different isoforms.	Lipid metabolism

RPS18	*Ribosomal protein S18.* Ribosomes, the organelles that catalyze protein synthesis, consist of a small 40S subunit and a large 60S subunit. Together these subunits are composed of 4 RNA species and approximately 80 structurally distinct proteins. This gene encodes a ribosomal protein that is a component of the 40S subunit. The protein belongs to the S13P family of ribosomal proteins. It is located in the cytoplasm. As is typical for genes encoding ribosomal proteins, there are multiple processed pseudogenes of this gene dispersed through the genome.	Ribosome<br>Proteasome<br>Ubiquitin
DRL1	*DRL1 protein. T*he DRL1 gene may encode a homolog of the Elongator-associated protein KTI12 of yeast, and is possibly a chromatin-associated protein.	Chromatin
**DECREASED (3 proteins)**		
*SMNDC1**	*Survival of motor neuron-related-splicing factor 30.* This is a nuclear protein that has been identified as a constituent of the spliceosome complex.	RNA<br>Splicing
*UBXN7**	*UBX domain-containing protein 7*. Its over-expression converts the scaffold protein CUL2 to its neddylated form and causes the accumulation of non-ubiquitylated HIF1α.	Transcription
PCBP2	*Poly(rC) binding protein 2*. The protein appears to be multifunctional. Along with PCBP-1 and hnRNPK, it is one of the major cellular poly(rC)-binding proteins. It contains three K-homologous (KH) domains which may be involved in RNA binding. Together with PCBP-1, this protein also functions as a translational coactivator It has also been implicated in translational control of the 15-lipoxygenase mRNA.	RNA binding<br>Translation<br>Metabolism

Table S7. A comparison of the two patient subsets identified in the unsupervised hierarchical cluster analysis based on AML cell viability after treatment with metabolic inhibitors (Figure 5B). The percentage of cell viability was determined for each of the 72 patients after 48-hour treatment with each metabolic inhibitor. Hierarchical clustering was thereafter done based on the percent reduction of viability after treatment, which identified two clusters; an upper cluster including 24 patients with a reduction of cell viability after inhibitor treatment (a strong proapoptotic effect), with the greatest anti-viability effect seen after treatment with 2DG, while the lower cluster of 48 patients showed a weaker reduction of viability (weaker proapoptotic effect) after inhibitor treatment. The table shows (from left to right) the inhibitor tested, the median relative viability (percent viability in inhibitor-treated cultures relative to the corresponding inhibitor-free control cultures) and the *p*-value (Mann-Whitney *U-test*). Differences were regarded as statistically significant when $p \leq 0.05$ (no correction for the number of comparisons, significant *p*-values shown in bold).

Metabolic inhibitor	Median viability (%) for inhibitor-treated cultures relative to inhibitor-free control cultures		
	**Upper cluster (24 patients)**	**Lower cluster (48 patients)**	***p*-value**
Metformin	92.6	95.9	0.076
2DG	57.9	90.3	**0.000**
6AN	85.1	93.9	**0.013**
BPTES	94.6	96.5	0.202
ST1326	91.9	94.5	0.761
Lonidamine	89.3	90.6	0.515
AZD3965	99.7	99.6	0.662

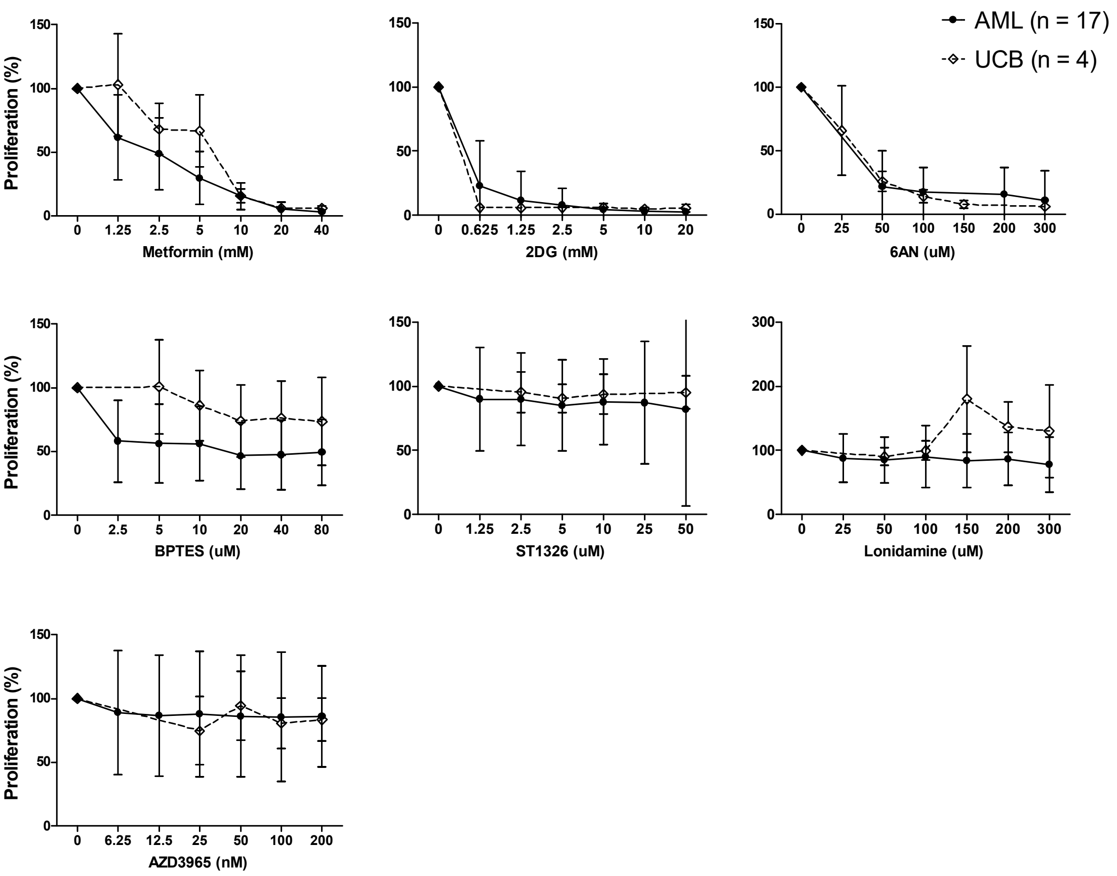

Figure S1. Initial dose-response studies of seven metabolic inhibitors; effects on the proliferation of primary human AML cells and umbilical cord blood (UCB) mononuclear cells. The [^{3}H]-thymidine incorporation assay was used to assess the effect of metabolic inhibitors on AML cell proliferation after 7 days of treatment. Primary AML samples derived from 20 patients were randomly selected from our biobank for these experiments; undetectable proliferation (< 1000 cpm) was found for three patient samples, therefore only results for the 17 patients with detectable leukemic cell proliferation are shown (solid line, filled circles). The umbilical cord blood (UCB) cells were derived from four donors (stippled line, open squares). The inhibitor concentrations are shown on the x-axis, while the y-axis shows percent proliferation after treatment compared to inhibitor-free controls (set as 100%). The inhibitor-response is presented as the mean response for all patients/donor samples +/- standard deviation.

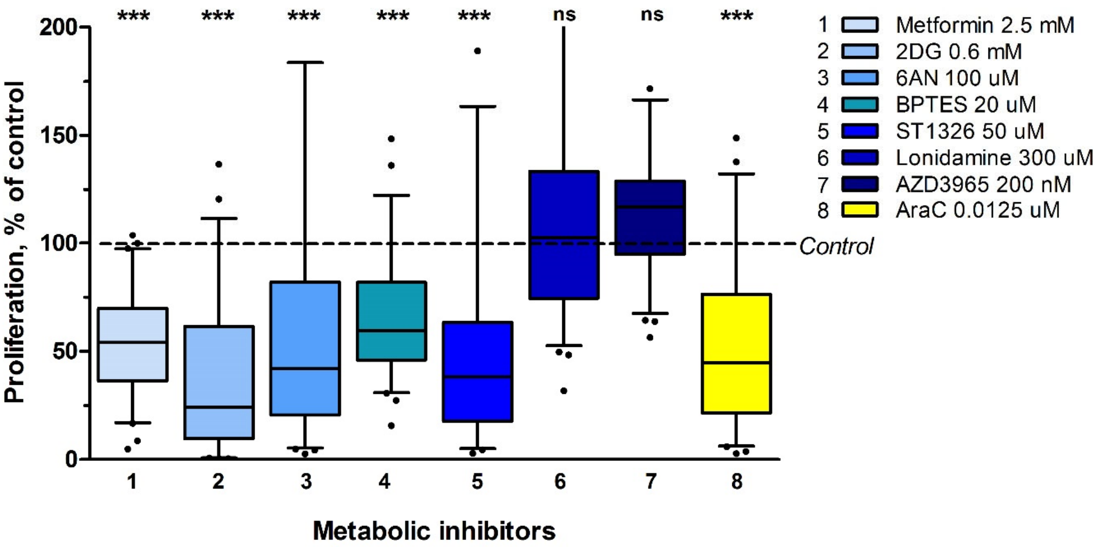

Figure S2. Effects of treatment with metabolic inhibitors or AraC on AML cell proliferation. AML cells were cultured alone in medium supplemented with cytokines or treated with metabolic inhibitors or AraC for 7 days in suspension cultures, before proliferation was measured using the [^{3}H]-thymidine incorporation assay. Detectable proliferation (defined as >1000 cpm) was found for 69 out of the 81 control cultures; while ST1326 was tested on 62 patients whereof 53 control cultures had detectable proliferation. The figure shows the results only for patients with detectable proliferation. The median proliferation (% of untreated controls) with 25/75 percentiles (5/95 percentile whiskers) is presented. The inhibitor-free (untreated) controls are set to 100% (stippled line). Statistically significant effects are shown after comparing inhibitor-treatment to the control cultures (ns, not significant, *** p-value < 0.0001). The Kruskal Wallis, with Dunn´s post-hoc test was used for statistical analyses.

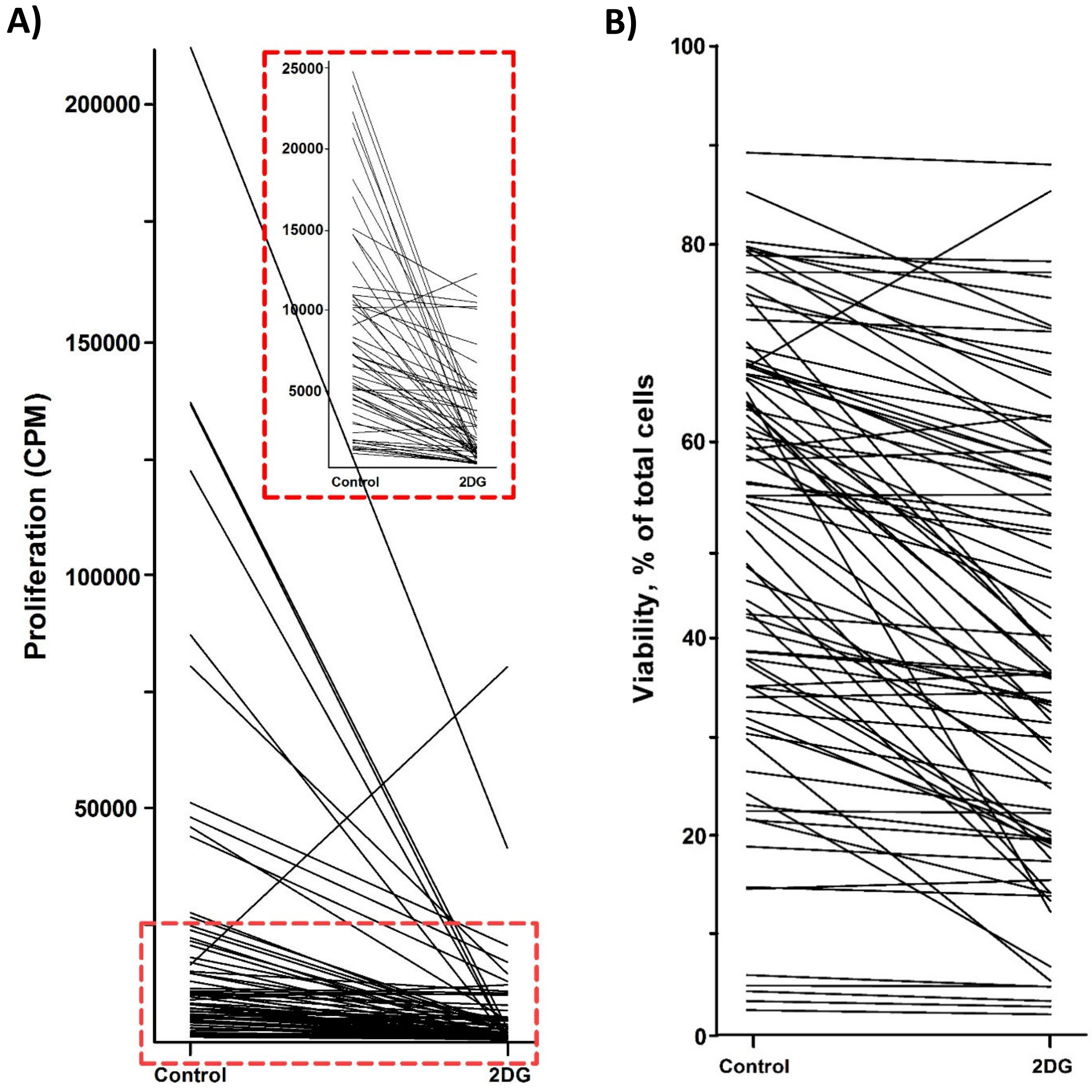

Figure S3. Effects of 2DG treatment on primary AML cell proliferation and viability. (A) AML cells were cultured for 7 days in suspension cultures in medium supplemented with cytokines either alone (controls) or treated with 0.6 mM 2DG before proliferation was measured using the [^{3}H]-thymidine incorporation assay. The data is shown as counts per minute (cpm) for individual patients, for the 69 out of 81 patients that showed detectable proliferation (defined as >1000cpm) in control cultures. The inset red stippled box shows the region displayed in higher magnification. **(B)** The AML cells were cultured for 48 hours with and without treatment with 0.6 mM 2DG before the percentage of viable cells was determined by flow cytometry (AnnexinV/PI assay). Percentage of viability was compared for leukemic cells cultured in medium alone (control) and treated with 2DG. The figure presents the results for the 72 patients with at least 5% viable cells in the control cultures.

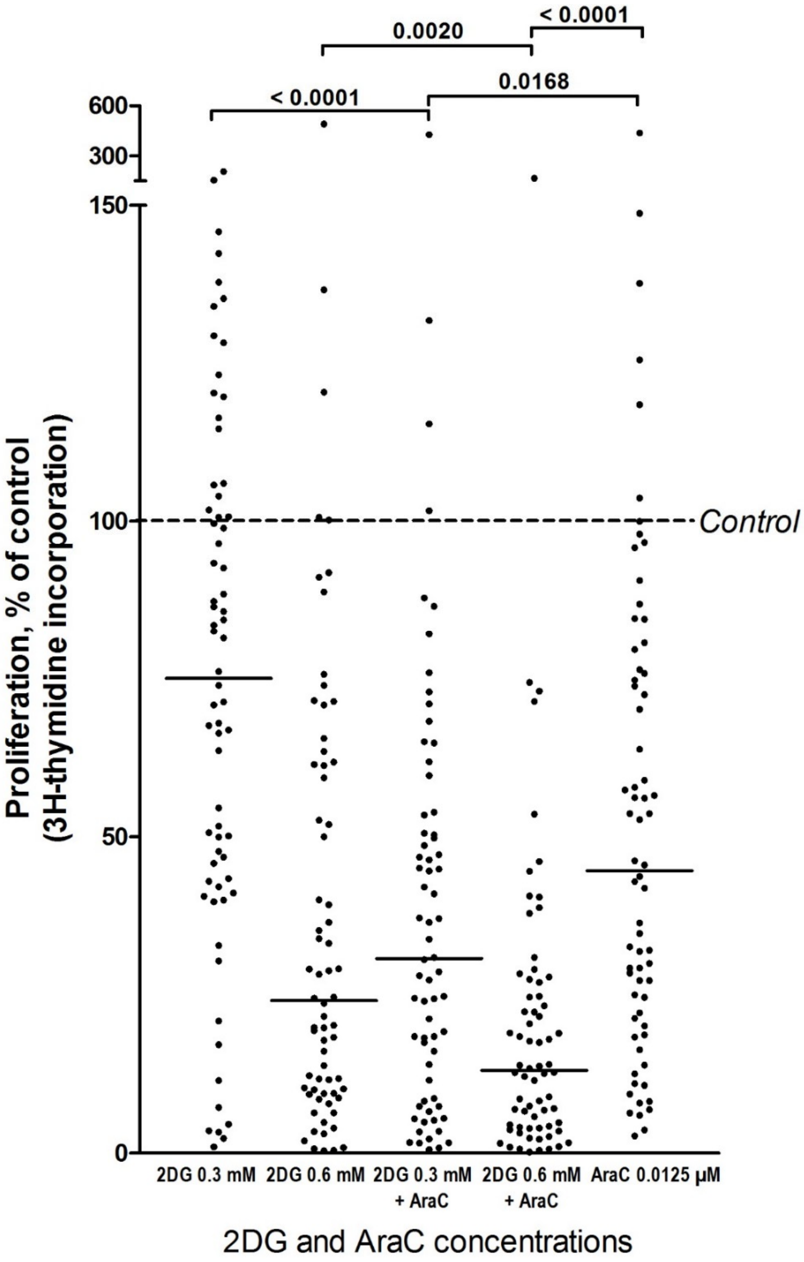

Figure S4. Effects of treatment with the glycolytic inhibitor 2DG or AraC on AML cell proliferation. AML cells were cultured for 7 days alone in medium supplemented with cytokines (control cultures) or treated with 2DG (0.3 or 0.6 mM), AraC (0.0125 μM) or a combination of 2DG and AraC, before proliferation was measured using the [^{3}H]-thymidine incorporation assay. Detectable proliferation was defined as >1000 cpm and was found for 69 out of the 81 control cultures; the figure presents the results for these 69 patients. The results are presented as the relative proliferation (% of untreated controls), and the median proliferation value is indicated as a solid line. The *p*-values (Mann Whitney *U*-test) for the significant statistical comparisons are shown at the top of the figure.

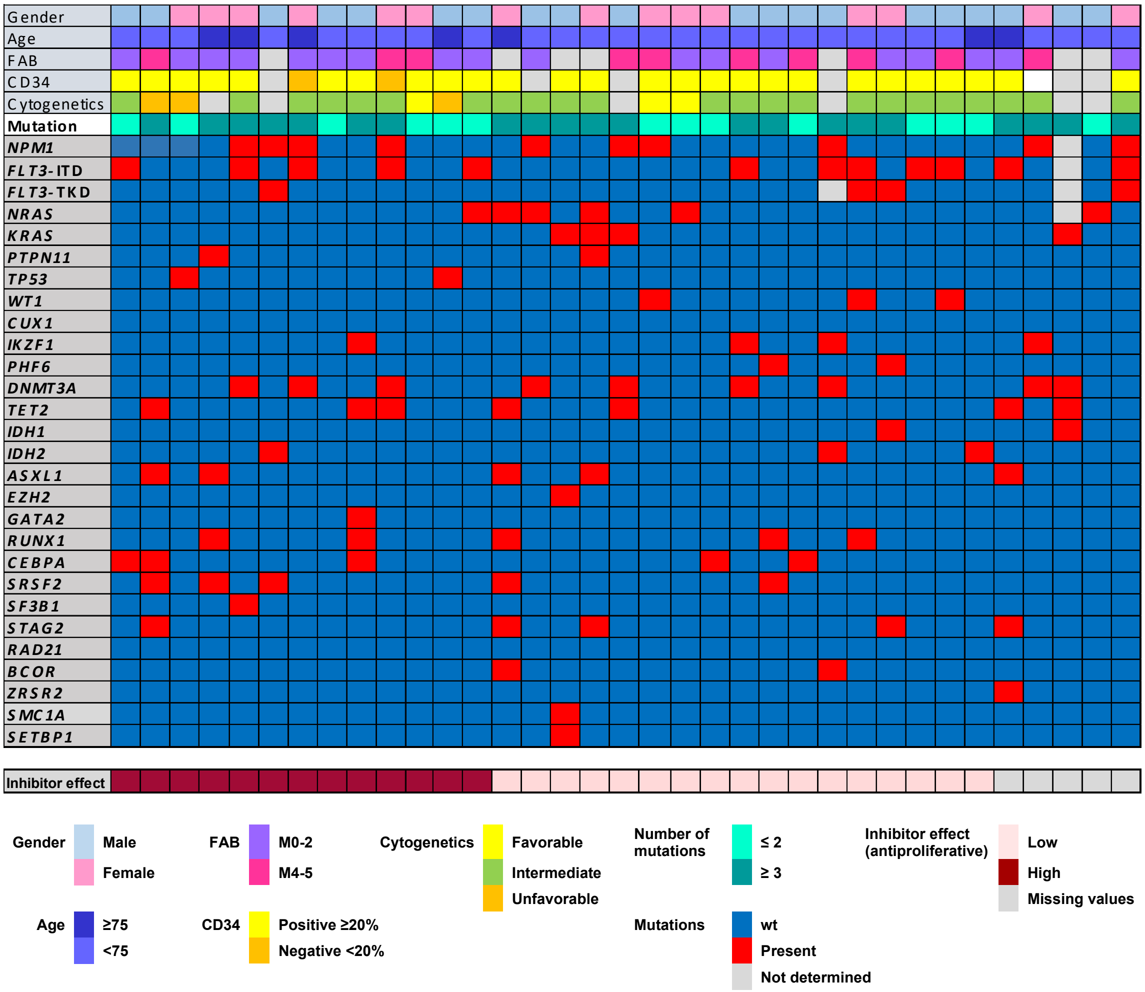

Figure S5. AML associated molecular genetics for 35 unselected patients (i.e. a consecutive subset from our patient cohort), a summary of the overall results. The mutations are listed to the left, where each column represents one patient and the presence of mutations are shown in red. Molecular genetics were analyzed for 35 patients, and as indicated in the bottom row (inhibitor effect), these 35 patients included 13 patients with a generally strong antiproliferative effect towards the metabolic inhibitors (dark red; based on Figure 3B, upper cluster of 29 patients), while 17 patients showed a weaker antiproliferative effect (light red, see Figure 3B), and the five last patients did not have detectable cytokine-dependent proliferation (shown in grey). The upper rows show patient gender, age, FAB classification (i.e. morphological signs of differentiation), CD34 expression and cytogenetics for each patient. A considerable heterogeneity can be seen between patients and patient groups with regards to molecular genetics. The 28 mutations can be classified into seven groups based on the main function of the encoded proteins: (i) intracellular signaling (FLT3-ITD/TKD, NRAS, KRAS, PTPN11), (ii) tumor suppressors (TP53, WT1, CUX1, IKZF1, PHF6), (iii) epigenetic regulation (DNMT3A, TET2, IDH1/2, ASXL1, EZH2, (iv) transcription factors (GATA2, RUNX1, CEBPA), (v) spliceosome (ZRSR2, SRSF2, SF3B1, BCOR), (vi) the cohesion complex (STAG2, RAD21, SMC1A) and (vii) others (NPM1, SETBP1) (see Papaemmanuil *et al*. [16]). The frequencies of mutations did not differ between the two main patient subsets/clusters that showed different responses toward metabolic inhibitors.

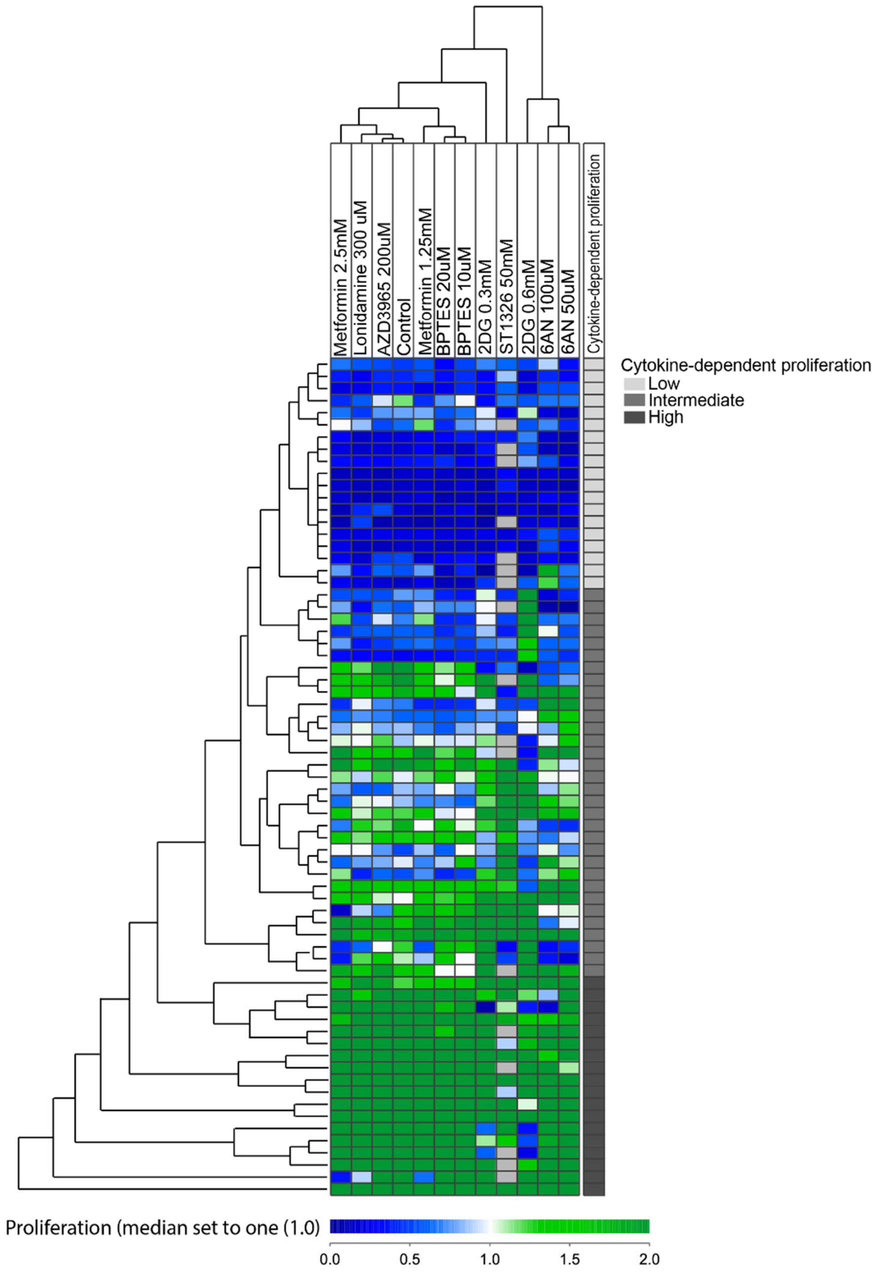

Figure S6. Hierarchical cluster analysis based on AML cell proliferation in cultures treated with and without metabolic inhibitors. Primary AML cells were cultured in medium supplemented with cytokines and proliferation was determined using the [^{3}H]-thymidine incorporation assay after 7-day treatment with various drugs and drug concentrations. An unsupervised hierarchical cluster analysis was performed based on the effects of all metabolic inhibitors and drug concentrations on AML cell proliferation. Results are shown for 69 out of the 81 patients with detectable proliferation in control cultures (defined as >1000 cpm). The proliferative response for each inhibitor was normalized to the corresponding median before analysis, thus green boxes indicate proliferation higher than median levels, while blue boxes are levels lower than median levels. The clustering of the metabolic inhibitors and their concentrations are shown at the top of the figure. We identified three main patient subsets: an upper subset generally showing low proliferation in all cultures compared to other patients (light grey), a lower subset showing generally high cytokine-dependent proliferation (dark grey color), and a large intermediate group with divergent effects (middle grey).

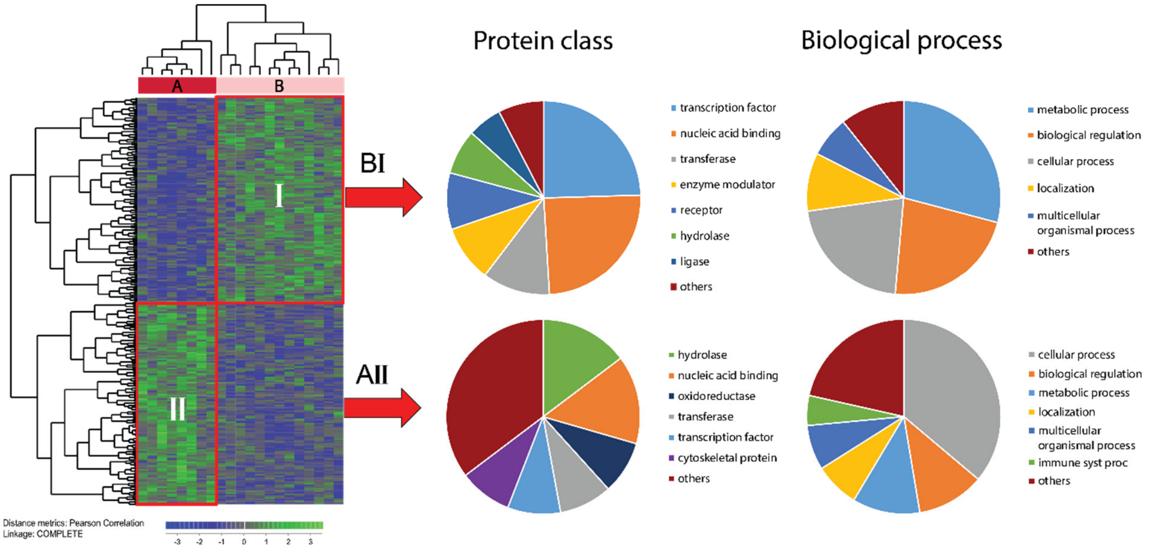

Figure S7. A comparison of global gene expression profiles for AML samples showing a strong antiproliferative effect towards metabolic inhibitors versus no or minimal antiproliferative effect (based on Figure 3B, upper and lower cluster, respectively). The global gene expression profiles were compared for 21 unselected AML patients that were available for analysis (i.e. the patients represent a consecutive subset in our cohort); eight of these patients had strong antiproliferative effects towards metabolic inhibitors (found among upper cluster of Figure 3B), while 13 patients had weaker antiproliferative or no effects (found among lower cluster of Figure 3B). A feature subset selection (FSS) analysis based on R-score > ±3.5 was performed which identified 265 differentially expressed (upregulated) genes. We further performed a hierarchical cluster analysis that separated both the genes (I and II, see cluster) and the patients (A and B) into two separate subclusters; subcluster BI had weak antiproliferative effects after treatment with metabolic inhibitors, while subcluster AII had strong antiproliferative effects after treatment with metabolic inhbitors. Based on the identified upregulated genes in each cluster, we performed gene ontology (GO) mapping based on the PANTHER database. By use of the categories Protein class and Biological process, we found several ontologies to be overrepresented in the two patient subclusters (shown in chart figures).

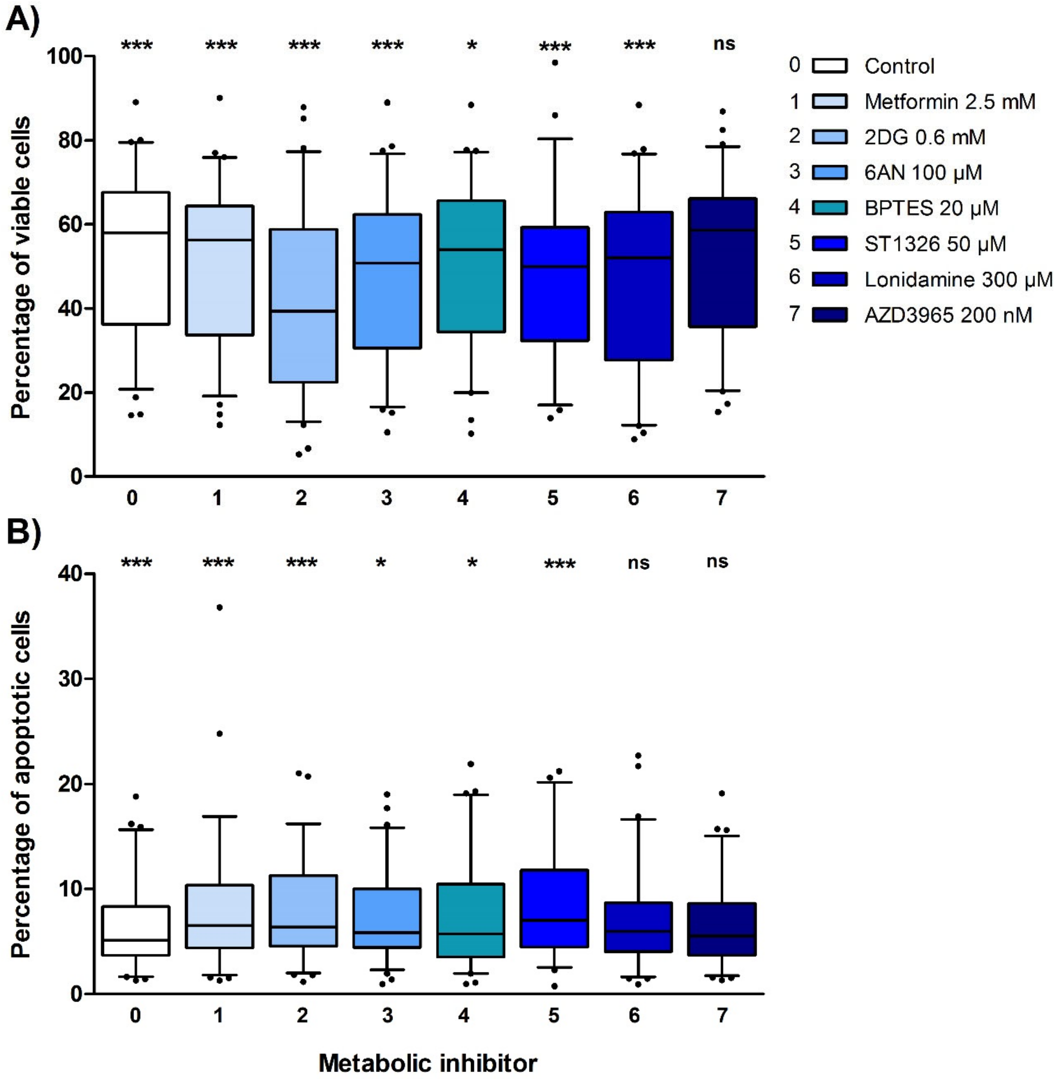

Figure S8. Effects of metabolic inhibitors on AML cell viability and apoptosis. AML cells derived from 78 consecutive patients were cultured for 2 days in suspension cultures in medium supplemented with cytokines with or without metabolic inhibitors, before the percentage of viable and apoptotic cells were determined by flow cytometry using the AnnexinV/PI apoptosis asssay. 72 patients had more than 5% viable cells in untreated control samples and were used in the statistical analysis. The percentage of **A)** viable and **B)** early apoptotic cells (median percentage, 25/75 percentiles, 5/95 percentile whiskers) is shown for all 72 patients. Significant effects of inhibitor treatment compared to untreated controls are shown. (Wilcoxon signed rank test, * p-value < 0.05, *** p-value < 0.0001; ns, not significant).

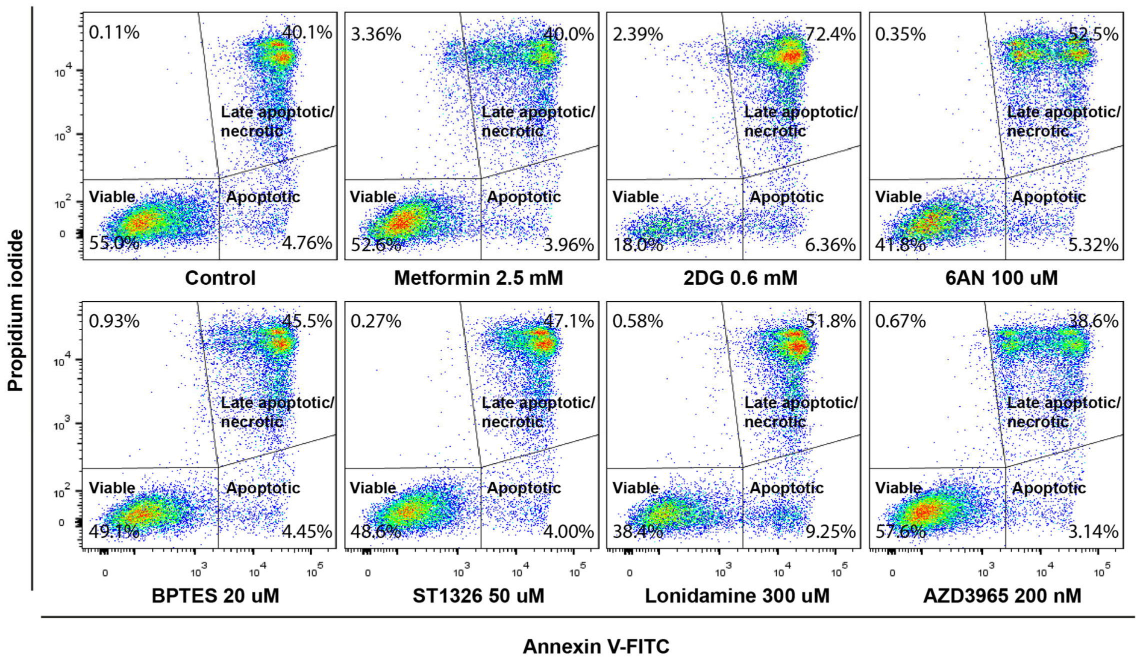

Figure S9. Flow cytometric analysis of AML cell viability; fraction of viable, apoptotic and late apoptotic/necrotic AML cells in cultures treated with or without metabolic inhibitors. These plots show the gating strategy used to define the percentage of viable, apoptotic and late apoptotic/necrotic cells, illustrated for one representative patient sample. The cells were incubated with medium alone (upper left panel, control), or treated with one of the seven metabolic inhibitors for 48 hours. Cell viability, necrosis and apoptosis were measured by flow cytometry using the ApoptestTM–FITC kit, and 10,000 events were collected and analyzed per sample.

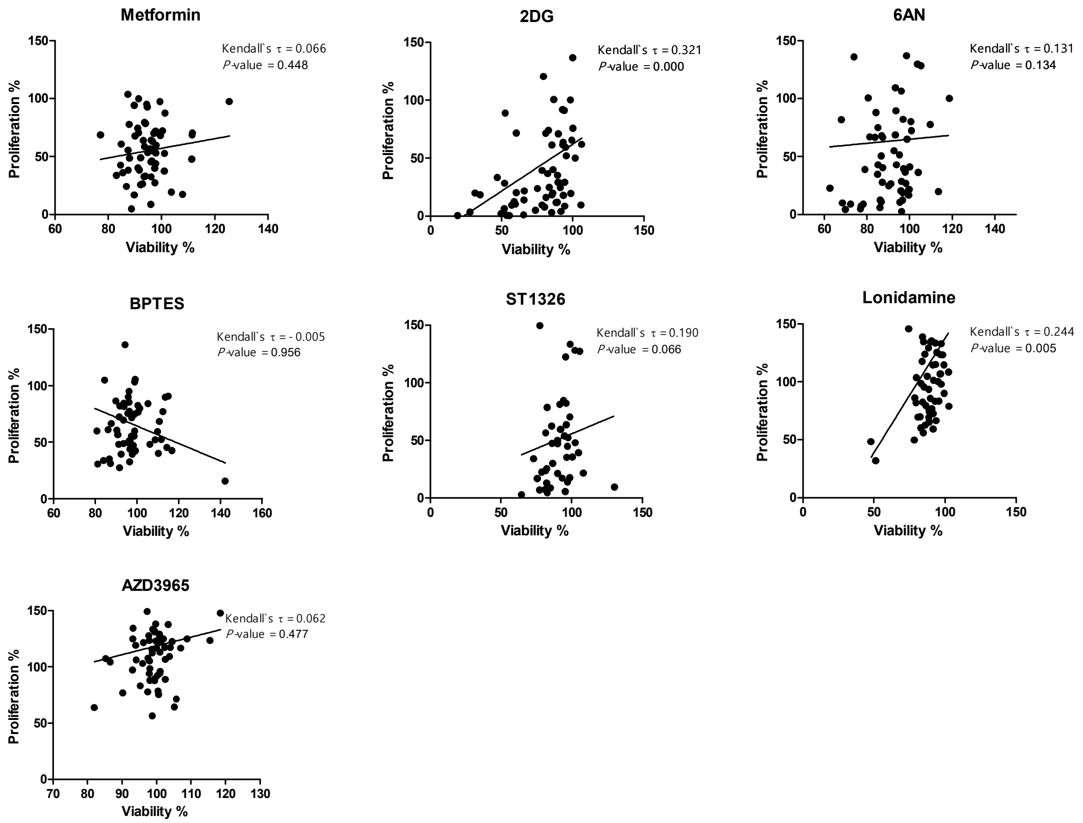

Figure S10. Antiproliferative and proapoptotic effects of metabolic inhibitors on primary human AML cells. We investigated whether the proapoptotic and antiproliferative effects of the various inhibitors on AML cells showed significant correlations. This analysis included all patients with detectable proliferation (i.e. corresponding to > 1000 cpm) and > 5% viable cells in control cultures. The correlation plots show the percentage of cytokine-dependent AML cell proliferation (y-axis) versus the percent of AML cell viability (x-axis) for each metabolic inhibitor (when compared to inhibitor-free control cultures). Kendall`s τ test was used for correlation analyses. The strength of the associations is presented as Kendall`s τ (-1 = 100% negative association, 1 = 100 % positive correlation), and the significance of correlation is presented as *p*-values (significant *p*-value < 0.05). Only 2DG and lonidamine showed significant, though weak, correlations between proliferation and viability (Kendall`s τ 0.321 and 0.244, and *p*-value 0.000 and 0.005, respectively).

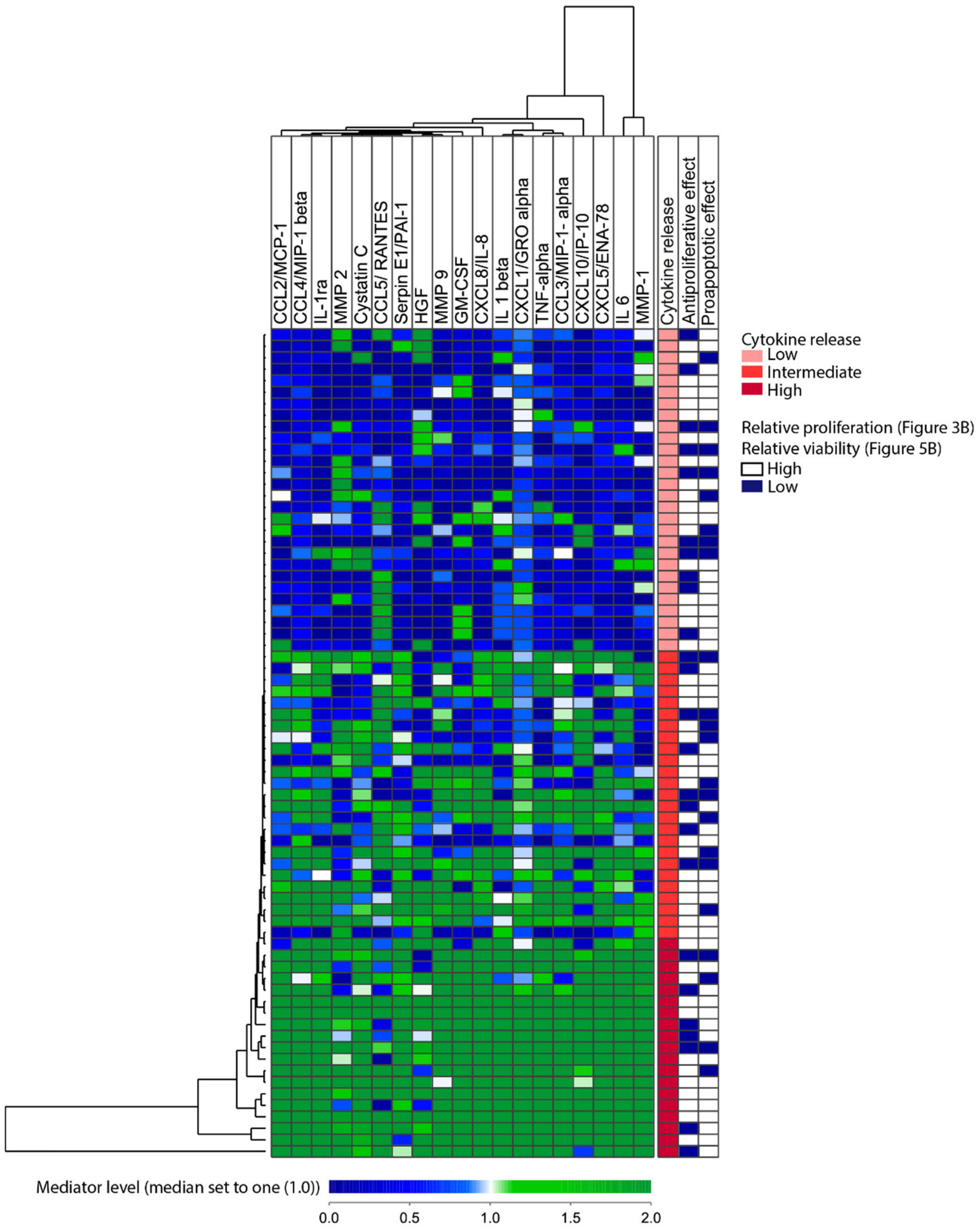

Figure S11. Unsupervised hierarchical cluster analysis of the constitutive release of soluble mediators by primary AML cells derived from 72 unselected patients, i.e. the patients with >5% viable cells after 48 hours of *in vitro* culture. The constitutive mediator release during 48 hours of *in vitro* culture was determined for 19 soluble mediators by multiplex assays. The concentrations were normalized to the corresponding median for each mediator before analysis. A low release of mediators is indicated by blue boxes, while green indicates higher release. Three patient subsets could be identified based on this cluster analysis (indicated by the column to the right of the cluster): an upper subset showing generally low release of mediators (light red), a lower subset with a high release of mediators (dark red) and a relatively large group of patients showing intermediate/divergent release (bright red, middle subset). Antiproliferative and proapoptotic effects after treatment with metabolic inhibitors showed no association with the capacity of constitutive mediator release (see right part of the figure; columns to the right are based on cluster analyses presented in Figure 3B and 5B).

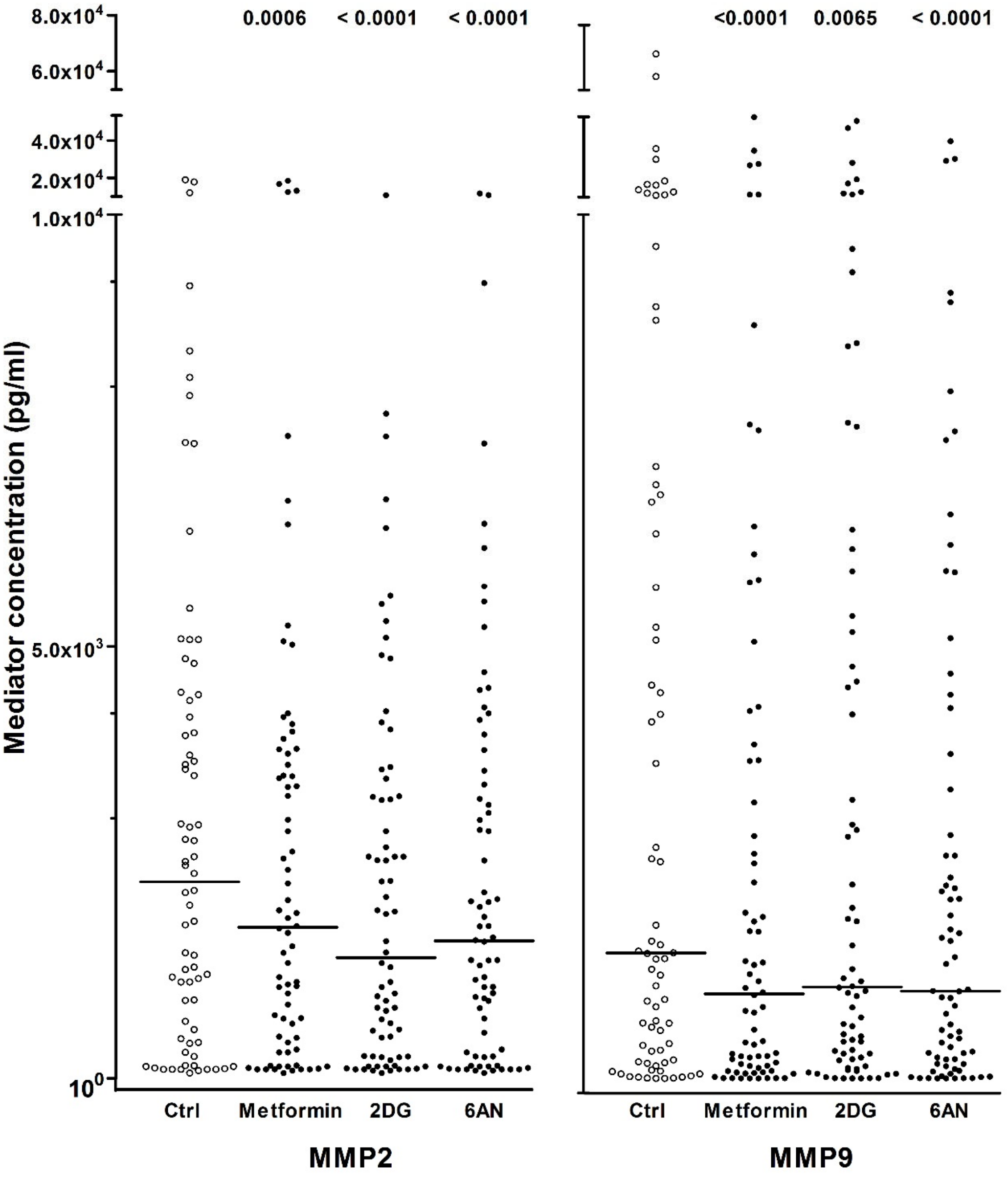

Figure S12. The constitutive release of MMP2 and MMP9 by primary human AML cells derived from 78 patients; effects of metformin, 2DG or 6AN. Primary human AML cells were cultured for 48 hours in medium alone (control cultures, ctrl) or treated with metabolic inhibitors before supernatants were harvested and supernatant levels of MMP2 and MMP9 were determined by Luminex analyses. The figure shows the mediator concentrations for each of 78 patients (i.e. the same 78 patients included in the viability analysis), and the median levels are shown as a solid line. The *p*-values for the comparisons of inhibitor-treated cultures versus the control cultures are indicated at the top of each figure (Wilcoxon's signed-rank test).

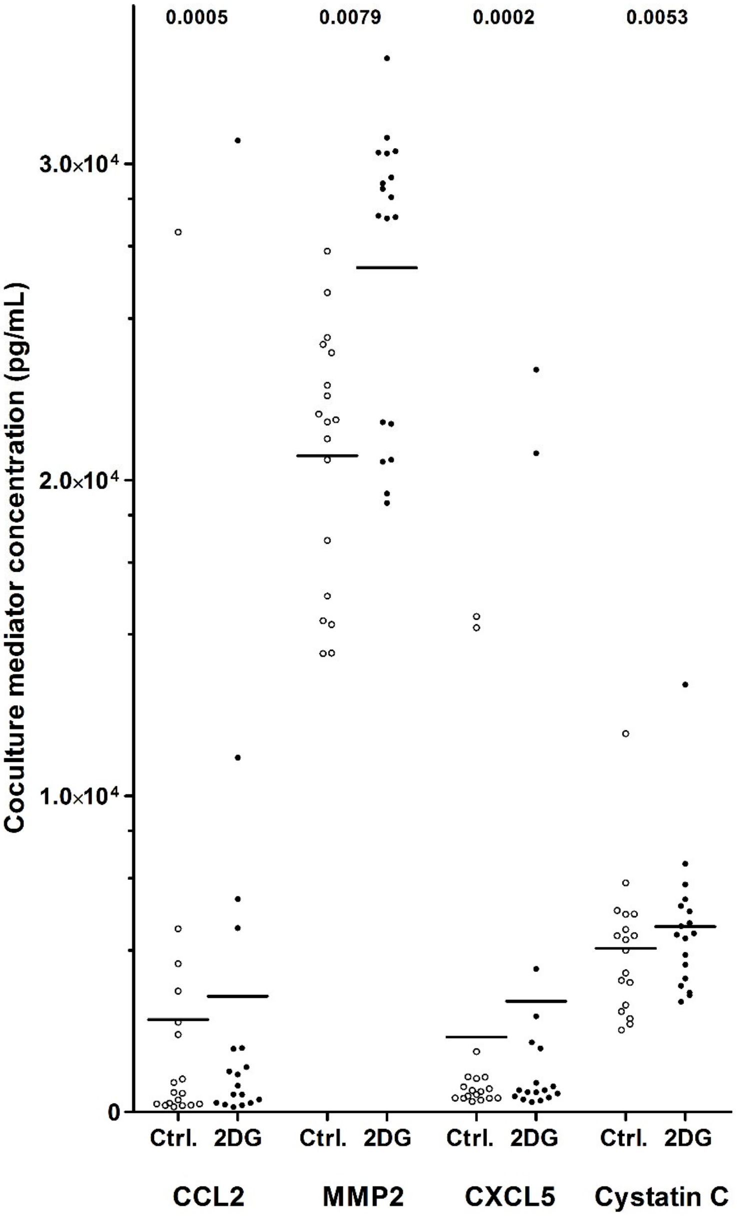

Figure S13. Effects of 2DG on supernatant mediator levels after transwell coculture of primary human AML cells and normal MSCs. The MSCs (one donor) and leukemic cells (derived from 18 patients) were cocultured in medium alone or treated with 0.6 mM 2DG for 48 hours, while separated by a semipermeable membrane during coculture. Supernatants were harvested and mediator levels were determined by Luminex analyses. The figure shows significantly altered levels of mediators (for four of the 19 mediators analyzed) after treatment with 2DG, for 18 randomly selected patient samples analyzed. Median levels are indicated as solid lines. The *p*-values for the comparisons of cultures prepared in medium alone (○) and with 2DG (●) are indicated at the top of the figure (Wilcoxon signed-rank test).

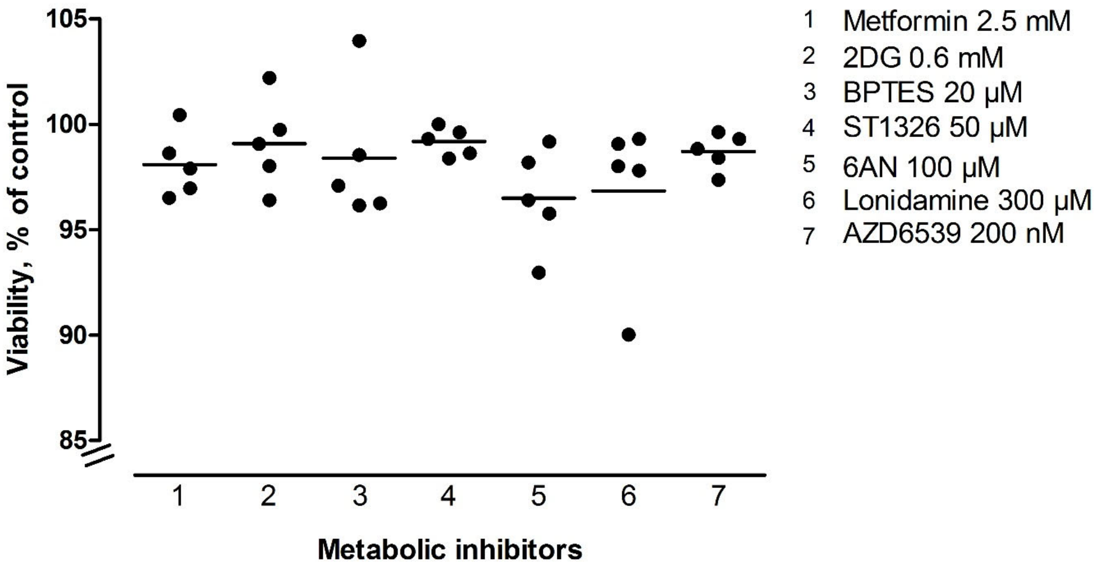

Figure S14. Viability of UCB cells derived from five donors after 48-hour treatment with metabolic inhibitors compared to untreated controls. UCB mononuclear cells were cultured for 48 hours in medium supplemented with cytokines with or without the presence of metabolic inhibitors. The percentage of viable cells for each UCB donor sample (i.e. viability of treated cultures relative to untreated control cultures) was determined by flow cytometry using the AnnexinV/PI assay. For each sample, 10,000 events were collected and analyzed. Solid lines indicate mean values.

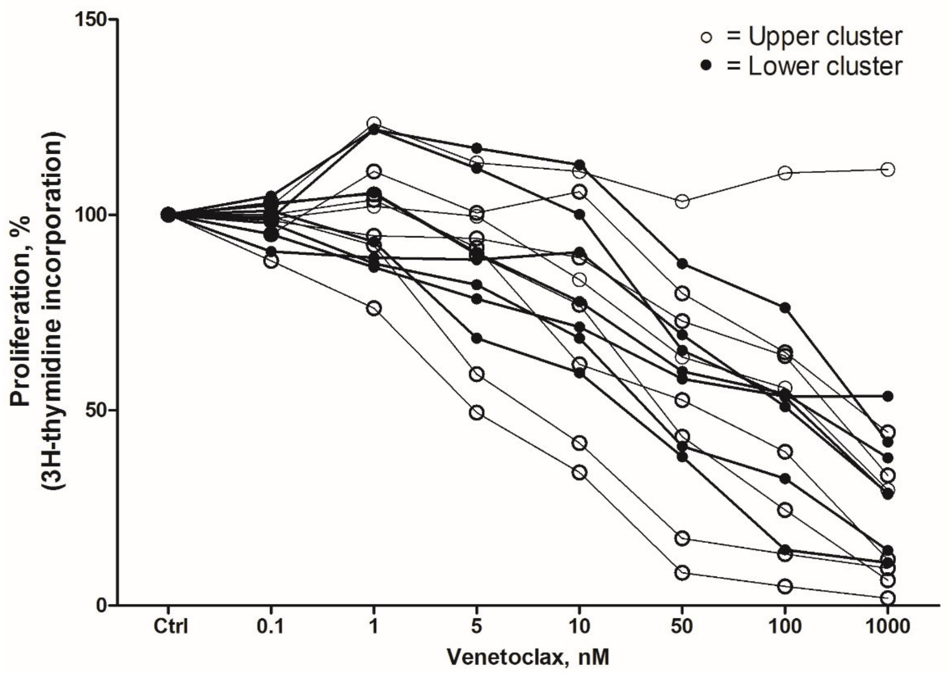

Figure S15. Effect of venetoclax on AML cell proliferation; a comparison of two patient clusters with different sensitivity towards metabolic inhibitors (Figure 3B). Primary AML cells were treated with a concentration range of venetoclax (0.1 - 1000 nM) for 7 days in medium supplemented with cytokines (G-CSF, SCF, FLT3L) before proliferation was determined using the [^{3}H]-thymidine incorporation assay. Results are presented as the relative proliferation of treated cultures compared to control cultures (% of untreated controls). Samples from sixteen AML patients were randomly chosen for analysis among the two patient subsets/clusters identified after hierarchical cluster analysis, shown in Figure 3B, that showed strong antiproliferative effects towards metabolic inhibitors (8 patients from upper cluster of 29 patients) and divergent/weaker antiproliferative effects toward metabolic inhibitors (8 patients from the lower cluster of 40 patients). One patient sample showed undetectable proliferation (defined as < 1000 cpm) and was excluded from the graph/analysis.

References

1. Bola, B.M.; Chadwick, A.L.; Michopoulos, F.; Blount, K.G.; Telfer, B.A., *et al.* Inhibition of monocarboxylate transporter-1 (MCT1) by AZD3965 enhances radiosensitivity by reducing lactate transport. *Mol Cancer Ther,* **2014**, *13,* (12) 2805-16.
2. Polanski, R.; Hodgkinson, C.L.; Fusi, A.; Nonaka, D.; Priest, L., *et al.* Activity of the Monocarboxylate Transporter 1 Inhibitor AZD3965 in Small Cell Lung Cancer. *Clin. Cancer Res.,* **2014**, *20,* (4) 926-937.
3. Sabins, H.S.; Bradley, H.L.; Tripathi, S.; Yu, W.M.; Tse, W., *et al.* Synergistic cell death in FLT3-ITD positive acute myeloid leukemia by combined treatment with metformin and 6-benzylthioinosine. *Leuk. Res.,* **2016**, *50,* 132-140.
4. Huai, L.; Wang, C.C.; Zhang, C.P.; Li, Q.H.; Chen, Y.R., *et al.* Metformin induces differentiation in acute promyelocytic leukemia by activating the MEK/ERK signaling pathway. *Biochem. Biophys. Res. Commun.,* **2012**, *422,* (3) 398-404.
5. Wang, F.F.; Liu, Z.F.; Zeng, J.S.; Zhu, H.Y.; Li, J.J., *et al.* Metformin synergistically sensitizes FLT3-ITD-positive acute myeloid leukemia to sorafenib by promoting mTOR-mediated apoptosis and autophagy. *Leuk. Res.,* **2015**, *39,* (12) 1421-1427.
6. Raez, L.E.; Papadopoulos, K.; Ricart, A.D.; Chiorean, E.G.; DiPaola, R.S., *et al.* A phase I dose-escalation trial of 2-deoxy-D-glucose alone or combined with docetaxel in patients with advanced solid tumors. *Cancer Chemother. Pharmacol.,* **2013**, *71,* (2) 523-530.
7. Chen, W.L.; Wang, J.H.; Zhao, A.H. A distinct glucose metabolism signature of acute myeloid leukemia with prognostic value (vol 124, pg 1645, 2014). *Blood,* **2014**, *124,* (18) 2893-2893.
8. Robustelli della Cuna, G.; Pedrazzoli, P. Toxicity and clinical tolerance of lonidamine. *Semin. Oncol.,* **1991**, *18,* (2 Suppl 4) 18-22.
9. Calvino, E.; Estan, M.C.; Simon, G.P.; Sancho, P.; Boyano-Adanez, M.D., *et al.* Increased apoptotic efficacy of lonidamine plus arsenic trioxide combination in human leukemia cells. Reactive oxygen species generation and defensive protein kinase (MEK/ERK, Akt/mTOR) modulation. *Biochem. Pharmacol.,* **2011**, *82,* (11) 1619-1629.
10. Parkhitko, A.A.; Priolo, C.; Coloff, J.L.; Yun, J.; Wu, J.J., *et al.*, Autophagy-dependent metabolic reprogramming sensitizes TSC2-deficient cells to the antimetabolite 6-aminonicotinamide, Mol. Cancer Res., 2014, pp. 48-57.
11. Chen, Y.; Xu, Q.; Ji, D.; Wei, Y.; Chen, H., *et al.* Inhibition of pentose phosphate pathway suppresses acute myelogenous leukemia. *Tumour Biol,* **2016**, *37,* (5) 6027-34.
12. Emadi, A.; Ju, S.A.; Tsukamoto, T.; Fathi, A.T.; Minden, M.D., *et al.* Inhibition of glutaminase selectively suppresses the growth of primary acute myeloid leukemia cells with IDH mutations. *Exp. Hematol.,* **2014**, *42,* (4) 247-251.
13. Seltzer, M.J.; Bennett, B.D.; Joshi, A.D.; Gao, P.; Thomas, A.G., *et al.* Inhibition of glutaminase preferentially slows growth of glioma cells with mutant IDH1. *Cancer Res.,* **2010**, *70,* (22) 8981-7.
14. Xiang, Y.; Stine, Z.E.; Xia, J.; Lu, Y.; O'Connor, R.S., *et al.* Targeted inhibition of tumor-specific glutaminase diminishes cell-autonomous tumorigenesis. *The Journal of clinical investigation,* **2015**, *125,* (6) 2293-2306.
15. Ricciardi, M.R.; Mirabilii, S.; Allegretti, M.; Licchetta, R.; Calarco, A., *et al.* Targeting the leukemia cell metabolism by the CPT1a inhibition: functional preclinical effects in leukemias. *Blood,* **2015**, *126,* (16) 1925-1929.
16. Papaemmanuil, E.; Gerstung, M.; Bullinger, L.; Gaidzik, V.I.; Paschka, P., *et al.* Genomic Classification and Prognosis in Acute Myeloid Leukemia. *N. Engl. J. Med.,* **2016**, *374,* (23) 2209-2221.
